# The Urinary Proteome Differs with the Presence and Type of Breast Cancer

**DOI:** 10.1021/acs.jproteome.5c00229

**Published:** 2025-11-03

**Authors:** Nur Aimi Aliah Zainurin, Russell M. Morphew, Alekhya Ganti, Dimitra Ivanova, Tim Gate, Helen Tench, Helen Phillips, Mandana Pennick, Luis A. J. Mur

**Affiliations:** † Department of Life Sciences, 1026 Aberystwyth University, Aberystwyth SY23 3DA, U.K.; ‡ Glan Clwyd Hospital, 1507 Betsi Cadwaladr University Health Board, Bodelwyddan LL18 5UJ, U.K.; § Wrexham Maelor Hospital, Betsi Cadwaladr University Health Board, Wrexham LL13 7TD, U.K.; ∥ Bronglais General Hospital, Hywel Dda University Health Board, Aberystwyth SY23 1ER, U.K.

**Keywords:** breast cancer, benign breast disease, symptom controls, proteomics, biomarkers, urine

## Abstract

Despite advancements in screening and treatment, the incidence of breast cancer (BC) and associated mortality are projected to increase. Therefore, developing a companion diagnostic for BC remains important. Herein, we explore the urinary proteome for biomarkers of BC: 130 urine samples from (1) newly diagnosed breast cancer (BC), *n* = 46, (2) benign breast disease (BBD), *n* = 36, (3) symptom control (SC), *n* = 30, and (4) healthy control (HC), *n* = 18. The BC class included preinvasive: ductal carcinoma in situ (DCIS) (*n* = 3), invasive ductal carcinoma (IDC) (*n* = 23), and IDC accompanied by DCIS (*n* = 8) classes. Protein profiling was performed using ThermoScientific ProteomeDiscoverer and analyzed using MetaboAnalyst v6.0, DAVID, and STRING v12.0. Analyses identified 346 significantly (*p* < 0.05) differentially expressed proteins (DEP) across BC, BBD, SC, and HC. Multivariate Receiver Operating Characteristic curves (five proteins) suggested Area Under the Curve values of 0.985, 0.989, and 0.999 distinguishing BC from BBD, SC, and HC, respectively. DEP elevated in BC included beta-glucuronidase isoform 1, fibrinogen gamma chain, alpha-actinin-1, peptidase inhibitor 16, cysteine-rich C-terminal protein 1 isoform X1, guanine nucleotide-binding protein G­(I)/G­(S)/G­(T) subunit beta-1, vascular cell adhesion protein 1, ATP-dependent translocase ABCB1, and tumor protein p63-regulated gene 1 isoform X1. BC types were differentiated based on calpain-2 and cystatin-C expression (*p* < 0.05). Thus, BC has distinct urinary–protein profiles based on clinical diagnosis, which could be used in real-time noninvasive BC monitoring.

## Graphical abstract

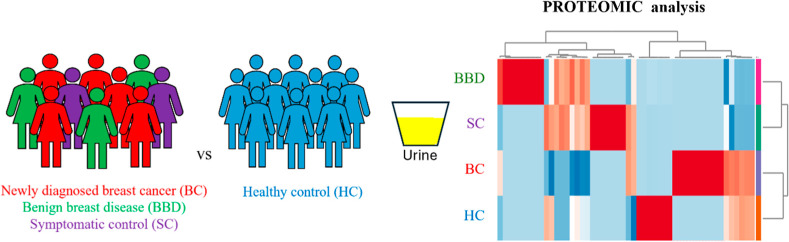

## 1 Introduction

Breast cancer (BC) remains a global health issue, ranking as one of the most prevalent and deadliest cancers in women.^1^ In the United Kingdom, about 1 in 8 females will develop BC in their lifetime.^2^ BC is a heterogeneous and multifactorial disease with a range of different types that vary in their morphology and clinical behavior. This makes BC challenging to treat and eradicate.^3,4^

Early detection is crucial for improving survival rates and favorable prognosis, as it often leads to more effective and less invasive treatment options. The current National Health Service Breast Screening Program (NHSBSP) in the UK is offered to women aged 50 to 70 triennially. The NHSBSP uses “rapid access” clinics based on the triple assessment strategy that has been the gold standard for evaluating breast symptoms.^5,6^ This involves a physical examination (examination of the breast for lumps or abnormalities by the clinician), imaging (breast scan using mammography, magnetic imaging resonance, ultrasound), and a biopsy (core needle biopsy or fine-needle aspiration cytology, an invasive approach to obtaining a small amount of breast tissue sample to be tested for cancer).^7,8^ Although this strategy has been successful in detecting many cases of BC at an earlier and more treatable stage, it has several limitations. In particular, there are many false positives among women with less or denser breast tissue mass than the normal, leading to the overtreatment of conditions that might not become life-threatening.^9^ Additionally, the interval between screenings allows for the potential development and progression of cancers.^10^ These limitations underscore the need for improved screening approaches and more personalized BC detection and prevention strategies.

In BC, hormone receptors (estrogen, ER and progesterone, PR) and human epidermal growth factor receptor-2 (HER2) are the most important histological markers associated with the prognosis of this disease.^11^ Expression patterns of receptors are used to classify BC into four main molecular subtypes, namely, luminal A, luminal B, HER2-enriched, and triple-negative BC, based on which subsequent treatment strategies will be applied.^12,13^ Such receptor expression patterns, or the proliferative marker (Ki67), are often defined by immunohistochemistry (IHC).^14,15^ Despite being relatively easy to perform, IHC has its limitations, especially reproducibility issues, due to subjective sampling and scoring of the results.^12^ These approaches could be improved through the development of new sensitive companion diagnostic platforms.

Recently, urinary proteins have been extensively explored as promising diagnostic and prognostic biomarkers for various cancers due to their noninvasive nature, cost-effectiveness, and ease of sampling and handling.^16–19^ Moreover, the changes in the urine composition can occur early in disease progression, potentially allowing for earlier detection of BC which makes it suitable for disease monitoring.^19^

In this study, we further explored proteomic approaches to detect changes in urine that could be linked to BC and not any other breast disease. We show statistically significant changes in the protein profiles in urine from BC patients that differ from samples from women with benign breast disease (BBD), symptom controls (SC) and, in comparison, to healthy controls (HC). If substantiated by further studies, such protein changes could be developed into a test to improve the overall outcomes in BC management.

## 2 Materials and Methods

### 2.1 Ethics Approval and Participant Recruitment

The study protocol was approved by the Health Research Authority (HRA) and Health and Care Research Wales (HCRW) (IRAS Project ID: 306872; CPMS study ID: 54143). The participants recruited in the study were female adults (age >18 years old) presenting with BC symptom(s) and suspected diagnosis of BC who went to rapid access clinics at Glan Clwyd Hospital and Wrexham Maelor Hospital, UK, and Bronglais General Hospital, UK, from September 2022 to February 2024. All consented females with BC symptoms underwent the same standard diagnostic procedures during their breast care clinic visit including physical examination of the breasts, mammography, and/or ultrasonography and/or needle core biopsy, as detailed by the American Joint Committee on Cancer staging.^20^ The baseline demographic data are given in Table 1, and Table 2 summarizes the clinical characteristics of the BC patients. Table S1 provides a fuller listing of the clinical metadata.

**Table 1:** Baseline Demographic Characteristics of the Participants (*n* = 130) in the Study^a^

sample groups		breast cancer (BC)	benign breast disease (BBD)	symptom control (SC)	healthy control (HC)	total
number of participants, *n*	overall	46	36	30	18	130
age range (mean ± SD)		35–92 (66.4 ± 12.90)	23–82 (50.3 ± 14.29)	23–72 (45.7 ± 15.58)	33–79 (50.3 ± 14.11)	23–92 (55.2 ± 16.42)
number of participants by age range, *n* (%)	<40	2 (4.35)	9 (26.47)	13 (46.43)	5 (27.78)	29 (23.02)
	40–49	3 (6.52)	9 (26.47)	3 (10.71)	4 (22.22)	22 (17.46)
	50–59	7 (15.22)	8 (23.53)	6 (21.43)	6 (33.33)	26 (20.63)
	60–69	15 (32.61)	4 (11.76)	3 (10.71)	1 (5.56)	21 (16.67)
	70–79	13 (28.26)	3 (8.82)	3 (10.71)	2 (11.11)	21 (16.67)
	>80	6 (13.04)	1 (2.94)	0	0	7 (5.56)
	not stated	0	2	2	0	4
BMI (kg/m^2^) (mean ± SD)		29.44 (±6.89)	28.21 (±6.06)	27.10 (±5.61)	25.87 (±5.35)	28.02 (±5.35)
smoking habits, *n* (%)	current	7 (15.22)	6 (16.67)	3 (10.0)	1 (5.56)	17 (13.08%)
	ex	14 (30.43)	13 (36.11)	10 (33.33)	1 (5.56)	38 (29.23%)
	never	25 (54.35)	17 (47.22)	17 (56.67)	16 (88.89)	75 (57.69%)
family history with BC, *n* (%)		19 (41.30)	17 (47.22)	8 (26.67)	2 (11.11)	46 (35.38%)
family history with other cancer type(s), *n* (%)		17 (36.96)	10 (27.78)	17 (56.67)	5 (27.78)	49 (37.69%)
history of breast disease, *n* (%)		5 (10.87)	11 (30.56)	1 (3.33)	0 (0)	17 (13.08%)
breast cancer symptoms (*n* = 112)	lump	41 (89.13)	23 (63.89)	20 (66.67)	NA	84 (75%)
	pain	1 (2.17)	8 (22.22)	11 (36.67)	NA	20 (17.86%)
	nipple discharge/changes	2 (4.35)	2 (5.56)	1 (3.33)	NA	5 (4.46%)
	others	3 (6.52)	5 (13.89)	2 (6.67)	NA	10 (8.93%)
	not stated	1 (2.17)	1 (2.78)	1 (3.33)	NA	3 (2.68%)

a Abbreviations: BMI: body mass index; SD: standard deviation; NA: not applicable.

**Table 2:** Clinicopathologic Characteristics of Breast Cancer Subjects (*n* = 46) in the Study

			*t*-test/ANOVA	fold change (FC = 2)	
clinicopathologic characteristics		values	p-value	sig. up	sig. down
age (years), mean ± SD (*n* = 46)		35–92 (66.4 ± 12.90)			
age groups, *n* (%)	31–50 years	6 (13.04)			
	51–70 years	21 (45.65)			
	>70 years	19 (41.30)			
BC side, *n* (%)	unilateral	left	23 (50.00)		
		right	16 (34.78)		
	bilateral	2 (4.35)			
	unknown	5 (10.87)			
type, *n* = 46 (%)	preinvasive	3 (6.52)	<0.05	180	13
	invasive	43 (93.48)			
	ductal carcinoma in situ (DCIS)	3 (6.52)	<0.05		
	invasive ductal carcinoma (IDC)	23 (50.00)			
	invasive lobular carcinoma (ILC)	3 (6.52)			
	invasive mucinous carcinoma (IMC)	3 (6.52)			
	invasive tubular carcinoma (ITC)	1 (2.17)			
	invasive papillary carcinoma (IPC)	1 (2.17)			
	mixed carcinoma	4 (8.70)			
	IDC accompanied by DCIS	8 (17.39)			
subtypes, IBC; *n* = 43 (%)	luminal A	30 (69.77)	>0.05		
	luminal B	3 (6.98)			
	HER2-enriched	1 (2.33)			
	triple negative	4 (9.30)			
	unknown	5 (11.63)			
grade (IBC, *n* = 34)	G1, *n* (%)	7 (20.59)	>0.05		
	G2, *n* (%)	16 (47.06)			
	G3, *n* (%)	11 (32.35)			
metastasis (IBC, *n* = 43)	present, *n* (%)	7 (16.28)	>0.05	12	70
	absent, *n* (%)	25 (58.14)			
	unknown, *n* (%)	11 (25.58)			
tumur focality (*n* = 43)	unifocal, *n* (%)	35 (81.40)	>0.05	33	33
	multifocal, *n* (%)	8 (18.60)			
tumur size (IBC, *n* = 43)	T1, *n* (%)	10 (23.26)			
	T2, *n* (%)	18 (41.86)			
	T3, *n* (%)	4 (9.30)			
	unknown, *n* (%)	11 (25.58)			
nodal status (IBC, *n* = 43)	N0, *n* (%)	15 (34.88)			
	N1, *n* (%)	11 (25.58)			
	N2, *n* (%)	2 (4.65)			
	N3, *n* (%)	2 (4.65)			
	unknown, *n* (%)	13 (30.23)			
Hormone Receptors (IBC, *n* = 43)					
estrogen (ER)	positive	35 (81.40)			
	negative	5 (11.63)			
	unknown	3 (6.98)			
progesterone (PR)	positive	29 (69.05)			
	negative	9 (21.43)			
	unknown	4 (9.52)			
human epidermal growth factor (HER2)	positive	5 (11.63)			
	negative	34 (79.07)			
	unknown	4 (9.30)			

### 2.2 Chemicals and Reagents

Analytical grade reagents and solvents used in the experiment were purchased from Sigma-Aldrich Chemicals (St. Louis, MO, USA), Fisher Scientific (Pittsburgh, PA), and Merck Chemicals (Darmstadt, FR Germany) unless otherwise stated. The solvents used for sample extraction and mass spectrometry (MS) analysis were of analytical grade (purity > 99%).

### 2.3 Sample Extraction and Preparation

Midstream 10–30 mL urine samples were collected in a sterile container and immediately stored in −80 °C for long-term storage until further analysis. For assessment, samples stored at −80 °C were thawed at 4 °C overnight. A total of 3 × 1 mL aliquots of the fully thawed samples were transferred to microcentrifuge tubes and centrifuged at 4200*g* for 5 min at 5 °C. Proteins were precipitated using 20% w/v ice cold trichloroacetic acid in acetone (1:1 [v/v] ratio), incubated at −20 °C for 1 h, and centrifuged at 18,000*g* for 15 min at 4 °C. The supernatants were discarded, and pellets were washed in 1 mL of ice-cold acetone. After that, the pellets were centrifuged at 18,000*g* for 10 min at 4 °C, acetone was discarded, and the washing step was repeated. After discarding the second wash, pellets were air-dried for 15–20 min at −20 °C allowing the complete evaporation of acetone. Dried protein pellets were resuspended in 52 μL of 6 M urea and 100 Mm Tris buffer (pH 8) aided by a 3 × 1 min period in a sonication bath (FB15052, Fisherbrand) at 4 °C, 50–60 Hz followed by cooling down on ice. Proteins were quantified using Bradford solution (Biorad, UK) following the manufacturer’s protocol and using bovine serum albumin (Sigma Pharmaceuticals Ltd.) as standard.^21,22^

From each sample, 15 μg of protein was diluted in 100 μL of digestion buffer (4 mg/mL ammonium bicarbonate in deionized water), and 15 mg/mL reducing reagent ((dithiothreitol (DTT); 100 mM, Thermo Scientific R0861) in digestion buffer) was added and incubated at 60 °C for 30 min. Next, 18 mg/mL of alkylating reagent ((iodoacetamide (IAA); Thermo Scientific Chemicals 122270250) prepared fresh in digestion buffer) was added. Once the samples were incubated in darkness for 30 min, 15 mg/mL DTT was added to neutralize IAA and for stopping the reaction. Subsequently, trypsin stock solution was prepared by dissolving 20 μg of lyophilized mass spectrometry grade protease trypsin powder (Thermo Scientific 90057) in 20 μL of 50 mM acetic acid, pH 3. The trypsin stock solution was then activated by diluting the stock solution with digestion buffer by 10-fold to a 0.1 μg/μL concentration. After that, the sample was digested with the activated trypsin stock solution at a 50:1 ratio. The samples were then incubated at 37 °C overnight (16 h) at 300 rpm and then stored in a −20 °C freezer until ready to run.

### 2.4 Liquid Chromatography-Tandem Mass Spectrometry and Protein Profiling

Protein samples prepared were analyzed using liquid chromatography-tandem mass spectrometry (LC-MS/MS) using an Orbitrap Fusion Tribrid mass spectrometer (Thermo Scientific), with EASY-Spray source coupled to an UltiMate 3000 RSLCnano system (Thermo Scientific) equipped with a Thermo Scientific EASY-Spray ES905 HPLC Column (C18; 75 μm × 750 mm, with 2 μm particle size). The gradient elution consisted of ultrapure water (18.2 MΩ) with 0.1% formic acid as eluant A, while 80% acetonitrile with 0.1% formic acid as eluant B. Liquid chromatography was performed with a flow rate of 200 nL/min starting with 3% eluant B, then increasing to 30% from 0.5 to 50 min, followed by 30% to 40% over 5 min, then increasing to 95% B over a further 15 min, and held for 10 min before equilibrating at 3% for 30 min.

Ions were generated with a source voltage of 1800 V in positive mode, with an ion transfer temperature of 275 °C. Standard peptide analysis parameters were used; parent ions were detected in profile mode in the 375–1500 *m*/*z* range in the orbitrap at a resolution of 120,000 and a maximum injection time of 50 ms in positive mode. MS2 data was collected in data-dependent acquisition mode including charge states of 2–7. Dynamic exclusion of masses was conducted for 20 s after initial selection for MS2. Ions were formed by fragmentation by collision-induced dissociation with a collision energy of 35%. Resulting ions were detected in the Ion Trap in centroid mode. The mass spectrometry proteomics data have been deposited to the ProteomeXchange Consortium via the PRIDE partner repository with the data set identifier PXD061502.^23^

### 2.5 Proteome Discoverer Database Searching

Proteomics data was analyzed using Proteome Discoverer (PD), Thermo Fisher Scientific version 2.5. The mass spectra were searched using both Mascot and Sequest HT database search engines. Mascot and Sequest HT workflow nodes were configured with setting parameters, including protein databases “Global Proteome Machine (GPM), common Repository of Adventitious Proteins (cRAP)”, and “Human genome assembly (GRCh38)”, enzyme name “Trypsin”, a maximum missed cleavage of 2, and minimum and maximum peptide lengths of 5 and 144, respectively. Simultaneously, the precursor mass tolerance was set to 10 ppm, while the fragment mass tolerance was set to 0.02 Da. The target false discovery rate (FDR) threshold was set at 0.01 for filtering both peptides and proteins.^24–26^ Protein quantification included a precursor ion quantifier within the feature mapper. The protein abundances were normalized prior to data mining.

### 2.6 Statistical Analyses

All statistical analyses were performed using an R-based MetaboAnalyst v6.0 platform.^27^ Data matrix generated in the PD software was transformed with the Log_10_ transformation and scaled with Pareto scaling to achieve a normal distribution. Analysis of variance (ANOVA) and *t*-test were performed to statistically analyze between the groupings and pairwise comparisons, respectively. Unsupervised and supervised approaches used Principal Component Analysis (PCA) and Partial Least Squared-Discriminant Analysis (PLS-DA), respectively. Diagnostic potential was assessed using receiving operating characteristic (ROC) curves. Pathway enrichment analysis was conducted to link key protein variables to biological functions using the Database for Annotation, Visualization and Integrated Discovery (DAVID) Functional Tool (https://david.ncifcrf.gov/summary.jsp), while the ancestor chart of the top Gene Ontology (GO) linked to biological processes was retrieved from QuickGO. The Search Tool for the Retrieval of Interacting Genes/Proteins (STRING) platform was used to generate a protein–protein interaction (PPI) network.^28,29^

## 3 Results

A total of 130 urine samples were collected from female adults recruited onto the BECA clinical study and stratified into four groups: (1) newly diagnosed breast cancer (BC), (2) benign breast disease (BBD), symptomatic control (SC), and healthy control (HC) (Table 1). Urinary proteins from each sample were isolated, trypsin-digested, and profiled using LC-MS/MS, and a total of 1235 proteins with 7069 peptides were identified across sample groups. After filtering with a Protein FDR Confidence set at 0.1%, removing contaminants (including keratin-associated proteins) and removing proteins that fell out of the minimum inclusion of 50% of replicates, a total of 540 proteins with 5159 peptides were taken forward for further analyses.

Partial least-discriminant analysis (PLS-DA) of the protein profiles demonstrated that urinary profiles could be clustered based on the clinical condition with minimal overlap (Figure 1a). Pairwise comparison between clinicals groups revealed a clear separation of urinary protein profiles between BC and BBD, SC, and HC (Figure 1b–d).

**Figure 1:**
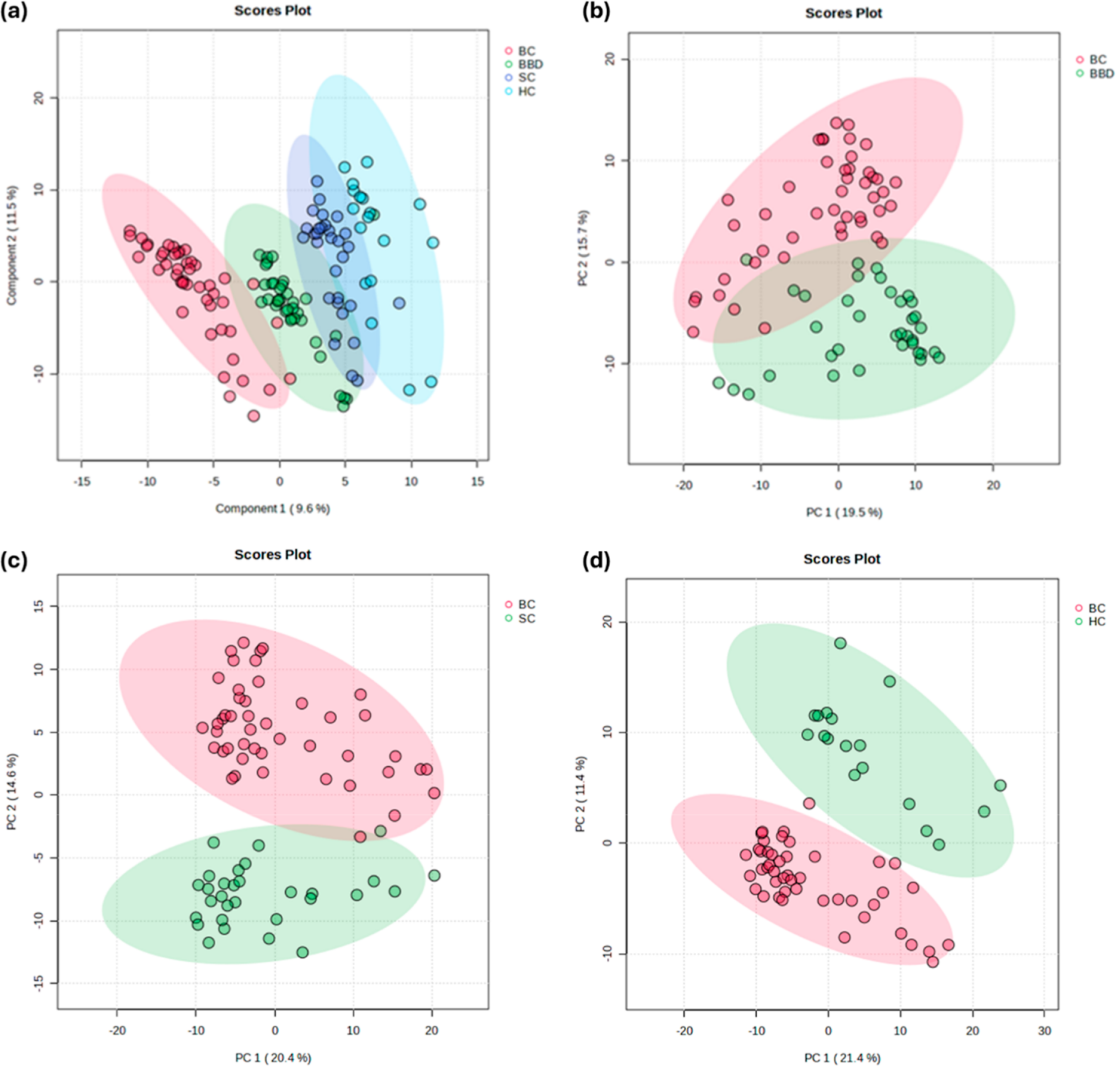
Partial least squared-discriminant analysis (PLS-DA) of urine proteomes from (a) volunteers with breast cancer (BC), benign breast disease (BBD), symptom control (SC), and healthy control (HC). Principal component analysis (PCA) based on pairwise comparisons of BC against (b) BBD, (c) SC, and (d) HC.

The major sources of variation among all groups were identified by ANOVA with *posthoc* analysis performed by applying Fisher’s LSD with a raw *P* value cutoff of 0.05. This suggested that there were 346 differentially expressed proteins (DEP) between the sample groups. The NCBI RefSeq of 346 proteins were converted to protein/gene ID using the SynGO platform^30^ (Table S2). These were compared to proteins that were annotated as human breast cancer-related genes in the UniProt database, of which 185 urinary proteins were identified and many had been previously identified in BC tissue, serum, and saliva (Table S3).^31–33^ Seven DEP (A1BG [alpha-1-B glycoprotein], AMBP [Alpha-1-Microglobulin/Bikunin Precursor], ANPEP [Alanyl Aminopeptidase], APOA4 [Apolipoprotein A4], C3 [Complement component 3], FGA (Fibrinogen Alpha Chain), and ORM1 [Orosomucoid 1]) had been linked to breast tissue and also found in urine (indicated with asterisks in Table S3).^32,34^ Although the remaining 161 DEP have not been linked to BC in previous studies, this suggested that urine samples could be used to report changes in breast tissue.

The top 50 DEP (ranked based on *P* values) are displayed using a column normalized average heat map (Figure 2). A heat map showing the levels in each sample is provided in the Supporting Information (Figure S1). This suggested 10 proteins, namely, beta-glucuronidase isoform 1 precursor (NP_000172.2), fibrinogen gamma chain isoform gamma-B precursor (NP_068656.2), alpha-actinin-1 isoform X9 (XP_011535567.1), peptidase inhibitor 16 precursor (NP_001186088.1), cysteine-rich C-terminal protein 1 isoform X1 (XP_011507958.1), guanine nucleotide-binding protein G­(I)/G­(S)/G­(T) subunit beta-1 isoform X1 (XP_047274005.1), lithostathine-1-alpha precursor (NP_002900.2), vascular cell adhesion protein 1 isoform a precursor (NP_001069.1), ATP-dependent translocase ABCB1 isoform 1 (NP_001335874.1), and tumor protein p63-regulated gene 1 protein isoform X1 (XP_024309245.1) (boxed in Figure 2), were more abundant in BC samples (*p* < 0.001) as compared to BBD, SC, and HC control groups.

**Figure 2:**
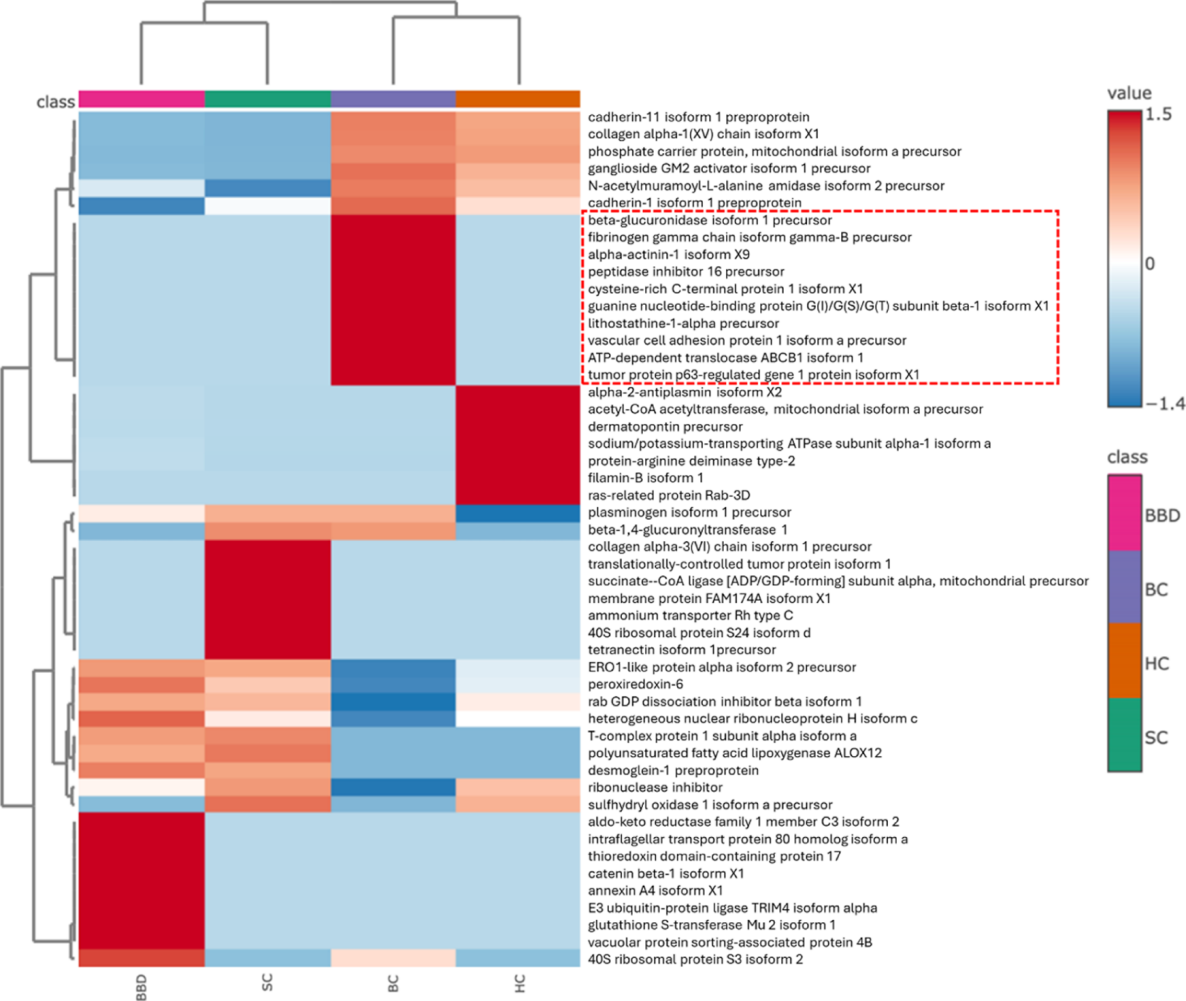
Heatmap analysis visualizing the differentially expressed urinary proteins (*p* < 0.001) (in rows) between breast cancer (BC), benign breast disease (BBD), symptom control (SC), and healthy control (HC) based on hierarchical clustering analysis applying Euclidean distance measure and Ward’s clustering method.

Following the approach shown in Figure 1b–d, pairwise comparisons of the urinary proteins were undertaken using Volcano plots (Figure 3a–c). These were based on a 2-fold change threshold and a false-discovery rate (FDR) of 0.05 threshold between sample groups: BC against BBD, SC, and HC data in turn. The number of targeted proteins is listed in Table 3 and identified in Table S4. Then, the diagnostic potential of the DEP targeted in each comparison was assessed (Figure 4). Multivariate assessments based on the top five sources of variation by the ROC curve suggested area under the curve (AUC) values of 0.985, 0.989, and 0.999 in discriminating between BC and BBD, SC, and HC, respectively. Univariate ROC curve assessments of the high AUC scoring proteins revealed that each had individual values of >0.8 (Table 4). There was some overlap with the proteins targeted by the pairwise and multivariate approaches, namely, fibrinogen gamma chain isoform gamma-B precursor, beta-glucuronidase isoform 1 precursor, alpha-actinin-1 isoform, X9 peptidase inhibitor 16 precursor, cysteine-rich C-terminal protein 1 isoform X, and guanine nucleotide-binding protein G­(I)/G­(S)/G­(T) subunit beta-1 isoform X1.

**Figure 3:**
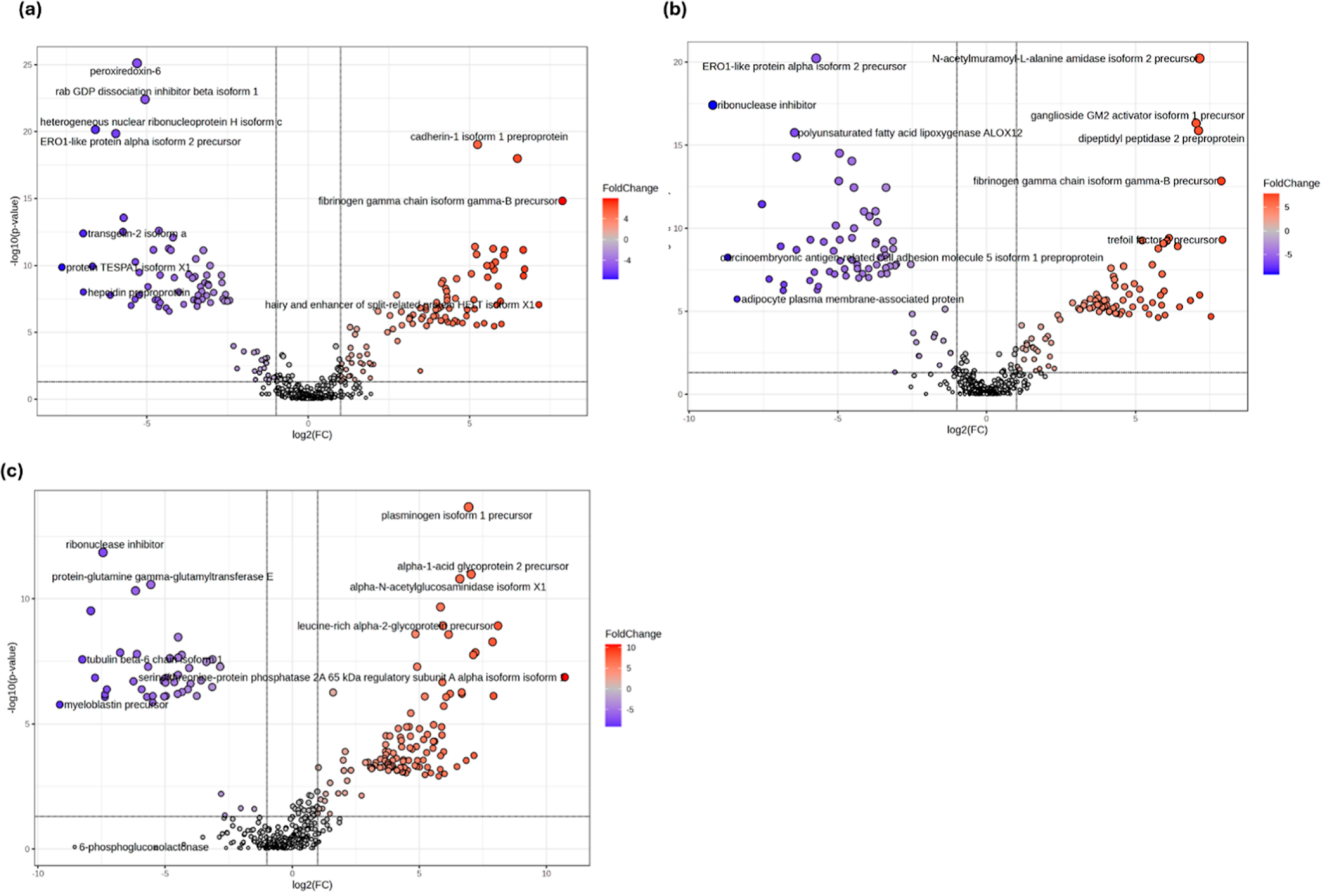
Volcano plots of urinary proteins that significantly upregulated (red) and downregulated (blue) in breast cancer (BC) compared with (a) benign breast disease (BBD), (b) symptom control (SC), and (c) healthy control (HC). Fold-change (FC) threshold 2 and false-discovery rate 0.05.

**Table 3:** Number of Differentially Expressed Proteins Based on Pairwise Comparisons between Breast Cancer (BC) and Benign Breast Disease (BBD), Symptom Control (SC), and Healthy Control (HC). Fold-Change (FC) Threshold 2 and False-Discovery Rate 0.05

comparison		differentially expressed proteins	increased	decreased
BC	BBD	220	107	81
	SC	193	98	75
	HC	185	121	46

**Figure 4:**
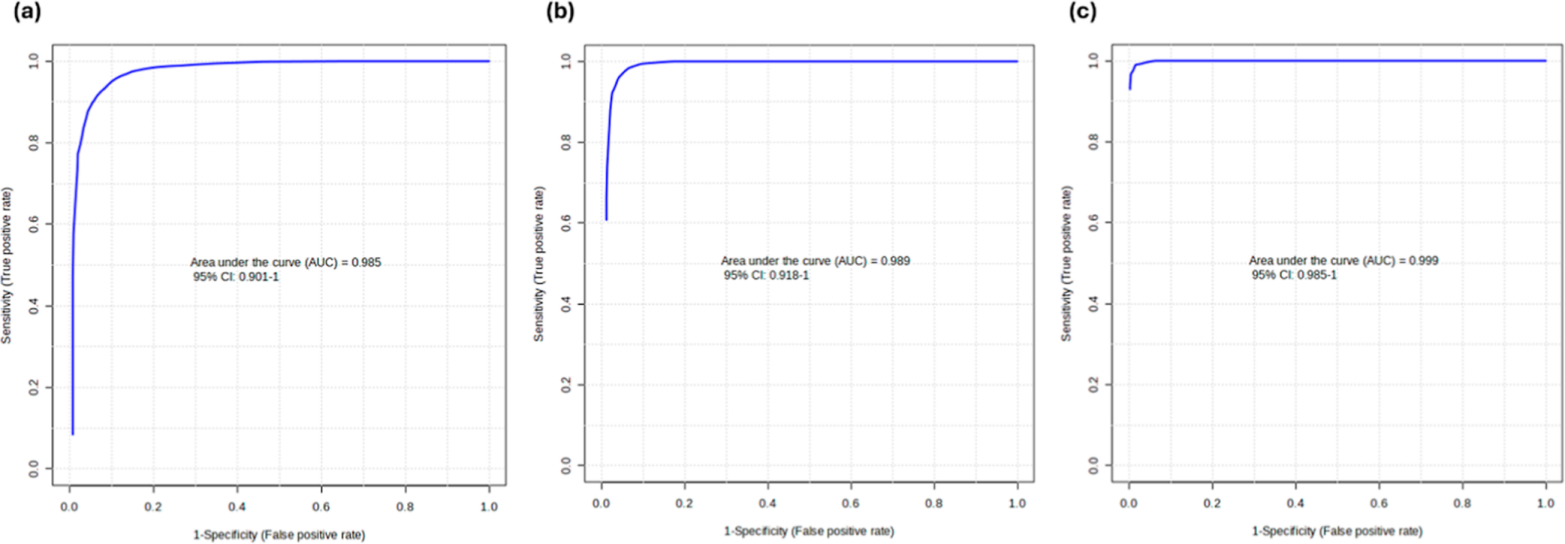
Multivariate Receiver Operating Characteristic (ROC) curve assessments comparing breast cancer (BC) and (a) benign breast disease (BBD), (b) symptom control (SC), and (c) healthy control (HC) groups. Models based on the top 5 sources of variation suggested area under the curve (AUC) values of 0.985, 0.989, and 0.999 in discriminating between BC and BBD, SC, and HC groups.

**Table 4:** Area under the Curve (AUC) of the Receiver Operating Characteristic (ROC) Curve Assessment of the Major Sources of Variation (*p* < 0.01) in Proteomic Comparison of Breast Cancer (BC against Benign Breast Disease (BBD), Symptom Control (SC), and Healthy Control HC Groups

		AUC (BC v)			(log2) fold-change (BC v)		
accession ID	protein	BBD	SC	HC	BBD	SC	HC
NP_068656.2	fibrinogen gamma chain isoform gamma-B precursor	0.92391	0.92391	0.92391	7.8822	7.8822	7.8822
NP_000172.2	beta-glucuronidase isoform 1 precursor	0.8587	0.8587	0.8587	5.2194	5.2194	5.2194
XP_011535567.1	alpha-actinin-1 isoform X9	0.88043	0.88043	0.88043	6.0527	6.0527	6.0527
NP_001186088.1	peptidase inhibitor 16 precursor	0.83696	0.83696	0.83696	5.563	5.563	5.563
XP_011507958.1	cysteine-rich C-terminal protein 1 isoform X1	0.82609	0.82609	0.82609	4.5996	4.5996	4.5996
XP_047274005.1	guanine nucleotide-binding protein G(I)/G(S)/G(T) subunit beta-1 isoform X1	0.81522	0.81522	0.81522	4.1736	4.1736	4.1736

The Database for Annotation, Visualization and Integrated Discovery (DAVID) Functional Tool was utilized to suggest the functions of the DEP (Table S5). The top 10 gene ontologies (GO) linked to biological processes, cellular locations, and molecular functions were identified (Figure S2). The top GOs in “biological processes” were “innate immune response” and “protein stabilization” (Figure S2a). A link between DEP and GO:0045087: innate immunity response was further suggested from an ancestor chart which was retrieved from QuickGO (Figure 5) based on 26 gene hits including complement genes (i.e., C1r subcomponent like (C1RL), factor I (CFI), C4A, and component 4 binding protein alpha (C4BPA)) and fibrinogen genes (i.e., alpha chain (FGA) and beta chain (FGB)). Both complement and fibrinogen genes are recognized for their vital role in the innate immune response contributing to host defense, inflammation, and immune regulation. While the complement genes are associated with immune cell activation, fibrinogen genes function as key inflammatory mediators in innate immunity.^35–40^ This suggested that the DEP could indicate increased stress that could reflect biotic stimuli. For cellular components, the “extracellular exosome”, “cytosol”, and “cytoplasm” were most prominent (Figure S2b). The predominant molecular function of GO was “protein binding” (Figure S2c).

**Figure 5:**
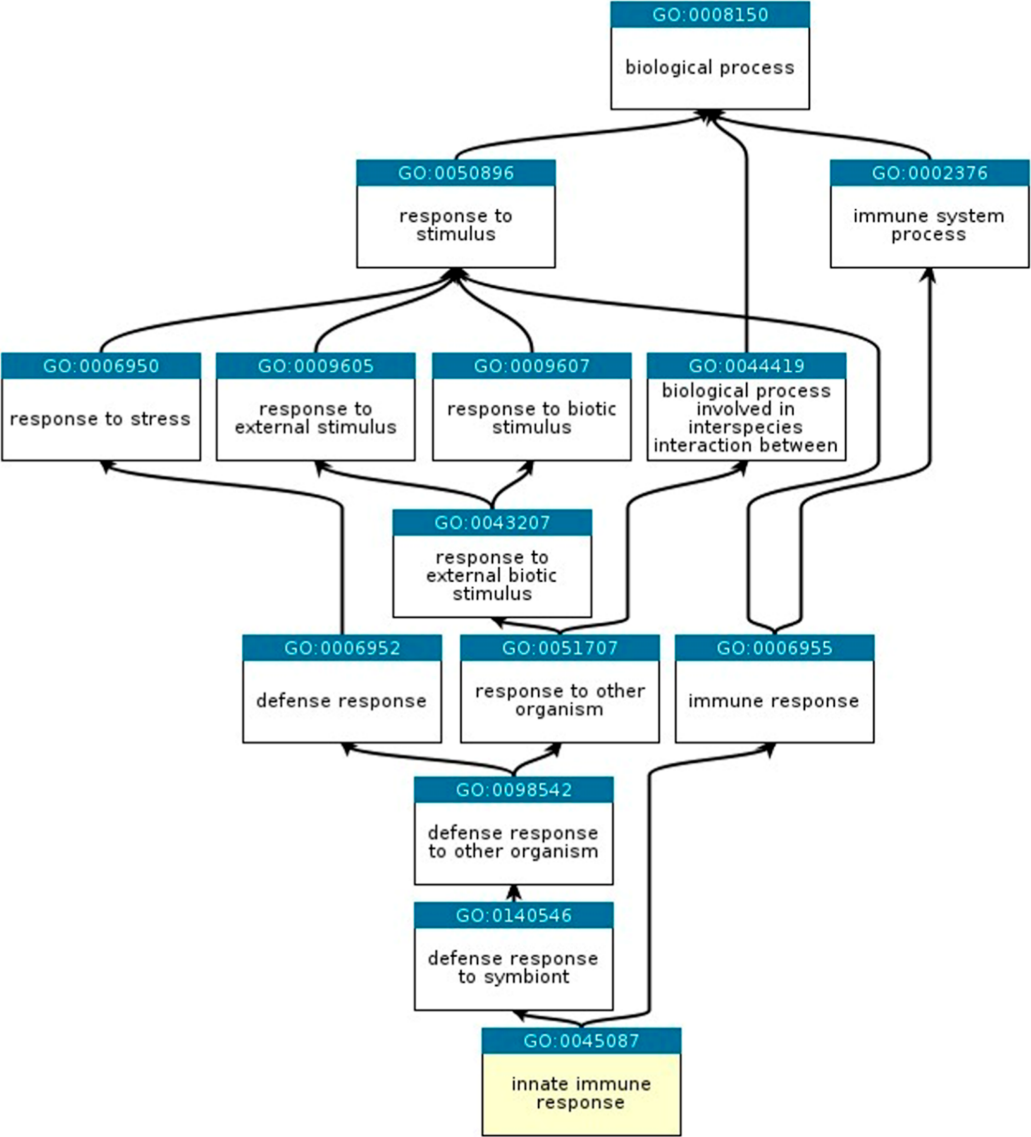
Ancestor chart for Gene Ontology (GO):0045087: innate immunity response retrieved from QuickGO and based on differentially expressed urinary proteins (*p* < 0.001) between breast cancer (BC) and benign breast disease (BBD), symptom control (SC), and healthy control (HC).

The protein–protein interaction (PPI) network of the DEP was analyzed through the Search Tool for the Retrieval of Interacting Genes/Proteins (STRING) platform.^28^ The full STRING network with five interactors and an interaction score of high confidence (0.90), FDR =< 0.05, was assessed. This associated the DEP into 15 clusters (Figure 6), and the membership of the top three clusters based on the number of proteins (designated “red”, “brown”, and “dark gold”) is listed in Table 5. The red cluster was the largest, and the constituent proteins were associated with nucleotide binding (GO:0032564, GO:0002135), the mucosa-associated IgA antibody (GO:0019862), and sulfonylurea receptor binding (GO:0017098). This suggested roles for the red cluster proteins in the immune response. Proteins in the brown cluster were associated with prostaglandin (GO:0047020) and steroid (GO:0045550) biosynthesis, which could suggest altered inflammatory responses. The “dark gold” cluster proteins were linked to GO:0003925, G protein activity which could align with roles in secretory extracellular exosome functions (Figure S2c).

**Figure 6:**
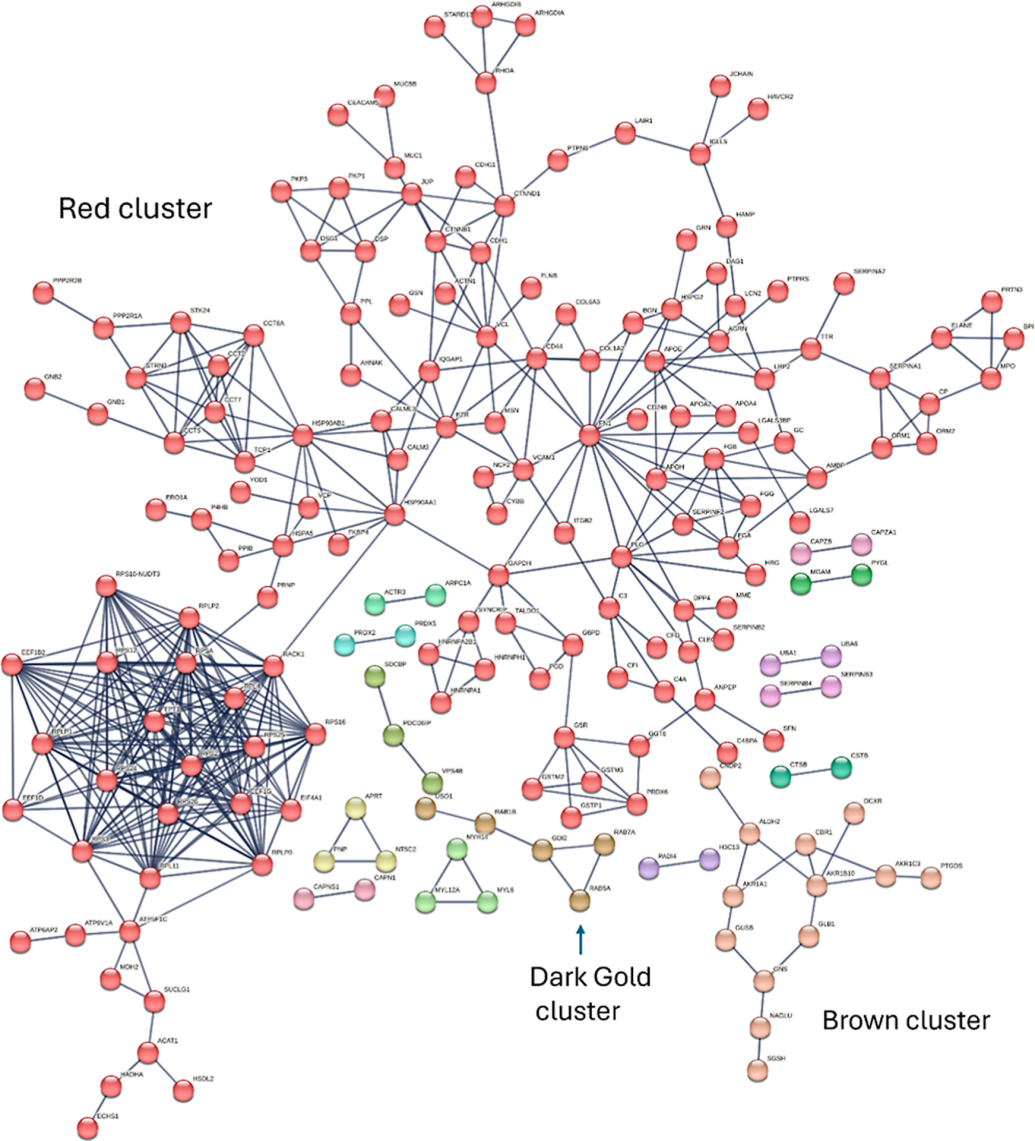
Protein–protein interaction (PPI) network based on differentially expressed urinary proteins (*p* < 0.001) between breast cancer (BC) and benign breast disease (BBD), symptom control (SC), and healthy control (HC).

**Table 5:** Clusters of Differentially Expressed Urinary Proteins (DEP) (*p* < 0.001) between Breast Cancer (BC) and Benign Breast Disease (BBD), Symptom Control (SC), and Healthy Control (HC) and Defined in Protein–Protein Interaction (PPI) Network (Figure 6)

cluster	protein names	molecular function
red (149 gene count)	ACAT1, ACTN1, AGRN, AHNAK, AMBP, ANPEP, APOA2, APOA4, APOE, APOH, ARHGDIA, ARHGDIB, ATP5F1C, ATP6AP2, ATP6 V1A, BGN, BPI, C3, C4A, C4BPA, CALM3, CALML3, CCT2, CCT5, CCT6A, CCT7, CD248, CD44, CDH1, CDH11, CEACAM5, CFD, CFI, CLEC3B, COL1A2, COL6A3, CP, CTNNB1, CTNND1, CYBB, DAG1, DPP4, DSG1, DSP, ECHS1, EEF1B2, EEF1D, EEF1G, EIF4A1, ELANE, ERO1A, EZR, FGA, FGB, FGG, FKBP4, FLNB, FN1, G6PD, GAPDH, GC, GGT6, GNB1, GNB2, GRN, GSN, GSR, GSTM2, GSTM3, GSTP1, HADHA, HAMP, HAVCR2, HNRNPA1, HNRNPA2B1, HNRNPH1, HRG, HSDL2, HSP90AA1, HSP90AB1, HSPA5, HSPG2, IGLL5, IQGAP1, ITGB2, JCHAIN, JUP, LAIR1, LCN2, LGALS3BP, LGALS7, LRP2, MDH2, MME, MPO, MSN, MUC1, MUC5B, NCF2, ORM1, ORM2, P4HB, PGD, PKP1, PKP3, PLG, PPIB, PPL, PPP2R1A, PPP2R2B, PRDX6, PRNP, PRTN3, PTPN6, PTPRS, RACK1, RHOA, RPL11, RPL4, RPLP0, RPLP1, RPLP2, RPS10-NUDT3, RPS12, RPS16, RPS2, RPS24, RPS25, RPS26, RPS3, RPSA, SERPINA1, SERPINA7, SERPINB2, SERPINF2, SFN, STARD13, STK24, STRN3, SUCLG1, SYNCRIP, TALDO1, TCP1, TPT1, TTR, VCAM1, VCL, VCP, YOD1	GO:0032564: dATP binding
		GO:0002135: CTP binding
		GO:0019862: IgA binding
		GO:0017098: Sulfonylurea receptor binding
		GO:0005094: Rho GDP-dissociation inhibitor activity
brown (13 gene count)	AKR1A1, AKR1B10, AKR1C3, ALDH2, CBR1, CNDP2, DCXR, GLB1, GNS, GUSB, NAGLU, PTGDS, SGSH	GO:0047020:15-hydroxyprostaglandin-D dehydrogenase (NADP+) activity
		GO:0045550: geranylgeranyl reductase activity
		GO:0047655: allyl-alcohol dehydrogenase activity
		GO:0008449: *N*-acetylglucosamine-6-sulfatase activity
		GO:0004032: alditol:NADP+ 1-oxidoreductase activity
dark gold	GDI2, RAB1B, RAB5A, RAB7A, USO1	GO:0003925: G protein activity

To further assess the potential diagnostic potential of urine proteins, BC samples were subclassified into separate groups: preinvasive (cancer cells remain confined to the ducts or lobules of the breast and have not invaded surrounding breast tissue) and invasive (cancer cells have spread beyond the ducts or lobules into surrounding breast tissue) (Table 2). Most of the BC patients (93%) were diagnosed with invasive breast cancer (IBC) of various types. Some samples were from metastatic IBC patients. The samples were further classified based on the relative expression patterns of key BC-associated receptors, ER, PR, and HER2. These were designated as samples from patients with luminal A (ER+, PR±, HER2−) (the major subtype), luminal B (ER+, PR±, HER2+), HER2-enriched (ER–, PR–, HER2+), and triple-negative (TNBC; ER–, PR–, HER2−) subtypes.

PLS-DA suggested that the urinary proteome could distinguish between preinvasive and invasive types, although based on a relatively low percentage of variation (7.2%) (Figure 7a). The key sources of variation were identified using ANOVA with *posthoc* analysis performed by applying Fisher’s LSD with a raw *P* value cutoff of 0.05. This identified calpain-2 catalytic subunit isoform 1 and cystatin-C precursor proteins as proteins whose different abundances could differentiate between BC types. In the mixed IDC-DCIS type samples, levels appeared to be similar to those with IDC alone (Figure 7 b). Next, a pairwise comparison of DCIS and IDC protein levels was undertaken using a Volcano plot (FC threshold = 2, *P* value threshold = 0.05). This targeted 40 proteins that are significantly higher and 3 that were lower in IDC compared to DCIS (Figure 7c). Besides the calpain-2 catalytic subunit isoform 1 and cystatin-C precursor protein, other proteins showed significant increases in IDC; prothrombin preprotein, endoplasmin (HSP90B1) precursor, and leucocyte-associated immunoglobulin-like precursor. These roles of proteins also result in such alterations in innate and adaptive immunity in the BC patients, most particularly in IDC cases.

**Figure 7:**
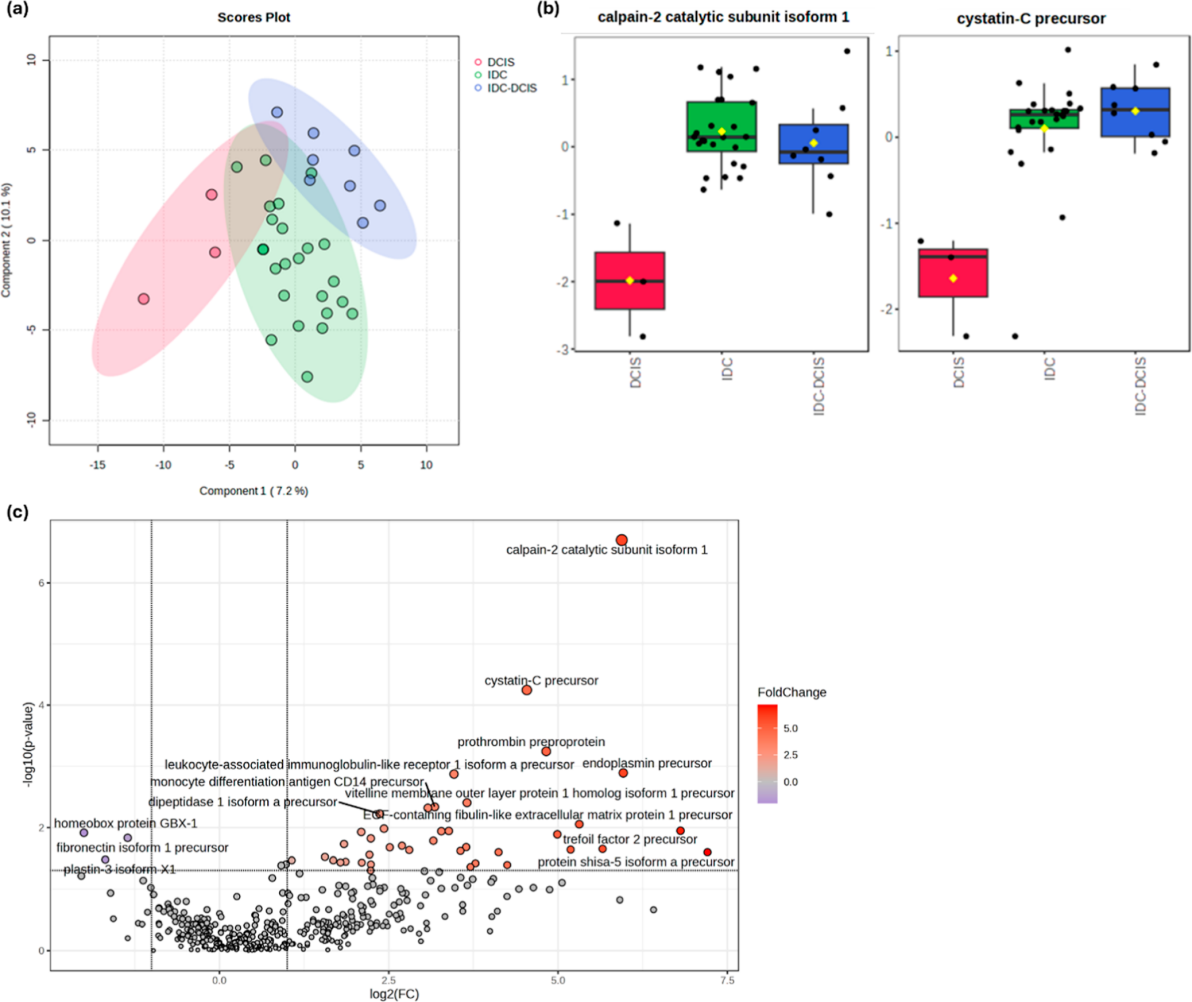
(a) PLS-DA of the urinary proteome of breast cancer (BC) types: ductal carcinoma in situ (DCIS) (preinvasive), invasive ductal carcinoma (IDC), and mixed IDC-DCIS. (b) Expression patterns of calpain-2 catalytic subunit isoform 1 and cystatin-C precursor in preinvasive cancer. (c) Volcano plot comparing IBC and DCIS with 40 significantly upregulated and 3 significantly downregulated proteins. FC threshold = 2, *P*-value threshold = 0.05.

## 4 Discussion

Breast cancer (BC) is a multifactorial disease, influenced by the interplay of biological, environmental, and lifestyle variations. As with all cancer, early detection is crucial for improving survival rates, but problems such as a reasonably high number of false positives remain an issue.^9^ In this current study, we sought to investigate the diagnostic potential of the urinary proteome for BC as well as new insights into the underlying carcinogenic pathologies.

### 4.1 Urinary Proteins Can Suggest the Presence of Breast Cancer

Assessments of the urine proteome identified statistically relevant changes in proteins linked to BC, BBD, and SC groups. Further, some of these changes had high AUC values (>0.9), suggesting that they have diagnostic potential. Other studies have observed differences in the urine proteome of BC patients.^34^ However, in no other study, to our knowledge, have comparisons been made between BC and BBD or SC classes. The inclusion of these comparisons in our analyses suggests that the proteins that we target are highly specific to BC. In line with this, the most important protein changes observed in BC samples were associated with tumorigenesis and tumor tissues. It may be that some 70% of the urinary protein population originate from the kidney and urinary tract, but our observations suggest that the remainder can include proteins that arise from the tumor microenvironment such as extracellular matrix proteins (e.g., COL1A2 and FN1), inflammatory and immune-related proteins (e.g., C3 and C4), and coagulation and fibrinolysis proteins (e.g., FGA, FGB, and FGG).^41–45^ As a result, urinary proteins could be used to detect not only BC but possibly responses to treatment.

Some of the targeted proteins appeared to be linked to the extracellular and structural components of the cell, namely, fibrinogen gamma chain isoform gamma-B and alpha-actinin-1. Detection of these cellular structural proteins in the urine proteome would suggest considerable shedding of these proteins into the circulatory system, possibly through cellular lysis or the production of extracellular vesicles in the tumor microenvironment.^46^

The fibrinogen (FG) molecule has three subunits called alpha, α (FGA), beta, β (FGB), and gamma, γ (FGG) and mainly synthesized by the liver and present in human plasma with a main role in hemostasis.^40,47^ Abnormal expression of FGG protein has been associated with bladder, hepatocellular, and pancreatic cancers^48–50^ as well as BC.^39,40^ FG is a component of the induced extracellular matrix (ECM) proteins, which has been recognized as an important molecule of BC metastatic cells.^51^ FGA, FGB, FGG, and FG (fibrin) were upregulated in the breast tissue of luminal B and TNBC subtypes, with FGA and FG (fibrin) being specifically associated with tissue-factor-induced thrombin signaling in cancer development.^39^

FGG expression was statistically correlated (*p* = 0.0065) with survival rates, and functionally, these were linked to RAS-MAPK signaling and associated in oncogene signaling, integrin signaling, and ECM remodeling.^52^ Li and colleagues^53^ also suggested ECM fiber-encoding genes, including FGG as the promising clinical prognostic biomarker for BC. ECM compounds including FGG are instrumental in modulating tumor cell behavior, governing cell proliferation and cell stemness/quiescence transition, through mechanical stimuli.^40,53^ Crucially, these changes in FGG proteins have been found only in breast tumor tissue and blood serum and have never before, prior to the current work, in urine samples.

Alpha-actinin-1 is a major component of the cytoskeleton molecule, which contributes to actin filament cross-linking proteins (F-actin).^54,55^ Via α-catenin links, F-actin interacts with integrin adhesions.^56^ α-Actinin-1 could be the prognostic marker for BC, specifically the basal-like (ER−) subtype. The elevated α-actinin-1 in mammary epithelial cells stimulates cell migration, which subsequently leads to reorganization of F-actin and E-cadherin. This major reorganization destabilizes cell–cell contacts, partially due to the increased tension at adhesion sites. Therefore, higher levels of α-actinin-1 (ACTN1) influence E-cadherin-based cell adhesions, leading to cancer progression through partial epithelial-to-mesenchymal transition (EMT).^54^ In BC, α-actinin-1 could also contribute to tumor metastasis by activating FAK/Src/JAK2/STAT3.^57^ Increased expression of the actinin-1 level has been linked with the Hippo signaling pathway.^58^ The Hippo signaling pathway controls organ size and tissue homeostasis, and its dysregulation contributes to tumorigenesis by promoting tumor growth via enhancing apoptosis.^59^ Roles for the Hippo pathway in BC include promoting EMT, stem cell generation, and metastasis.^60^

Other cytosolic proteins that were identified by our analyses have oncogenic roles. Guanine nucleotide-binding protein beta-1 (GNB1) is a component of heterotrimeric guanine nucleotide-binding proteins (G-proteins), and this class of signaling components are linked to cancer hallmarks and in establishing their tumor microenvironment.^61^ Overexpression of GNB1 was also observed in cervical squamous cell carcinoma, lung cancer cells, and hepatocellular carcinoma (HCC) tissues as opposed to adjacent normal cells and tissues.^49,62,63^ Within the context of BC, GNB1 expression was significantly higher in cancer tissues compared to normal glandular tissues in paired samples. Moreover, GNB1 expression increased with tumor, nodal, metastasis (TNM) stage (TNM1 vs TNM2/3/4), tumor grade (grade 2 vs 3), in ductal tumors and was associated with adverse patient outcomes (mortality vs disease-free survival).^64^ GNB1 expression may have a significant role in the antiapoptosis pathway mediated by the mammalian target of rapamycin (mTOR).^64^ Equally, recurrent mutations in GNB1 confer cytokine-independent growth and activate canonical G protein signaling.^65^ Interestingly, in other contexts, GNB1 was downregulated in cancer tissue, specifically in clear-cell renal carcinoma (ccRC) tissue, compared with normal kidney^66^ and colorectal cancer (CRC).^67^ Therefore, the role of GNB1 may be context-specific.

Peptidase inhibitor 16 protein (PI16) has been implicated in many cancers,^68^ including BC.^69^ In the present study, PI16 was more abundant in the urinary proteome of BC cases relative to BBD, SC, and HC. However, transcriptomic analysis suggested that PI16 was lower in tumor (*n* = 25) than in normal breast tissues (*n* = 25). This decrease was significant in the tumor tissues of early onset BC patients. Further, PI16 expression was negatively correlated with the overall survival of TNBC patients (*n* = 404) although not with other subtypes (luminal A, luminal B, and HER2+).^69^ Therefore, the increased expression in PI16 in the urinary proteome could reflect changes in posttranslational PI16 processing, increased secretion, or wider changes that are not occurring in BC tissues. Against this and more in line with our observations, increased expression of PI16 has been observed in other cancers, for example, hepatocellular carcinoma (HCC). Overexpression of PI16 in HCC tissue did not affect cell proliferation, migration, or invasion but was linked to poor survival rates.^68^ With pancreatic ductal adenocarcinoma (PDAC), overexpression of PI16 promotes proliferation through 2′-5′-oligoadenylate synthetase-like (OASL) activity.^70^ Interestingly, high OASL mRNA expression was correlated with favorable overall survival among HER2-overexpressing BC patients.^71^

A further contrast between the literature and our observations was seen with the cysteine-rich C-terminal 1 protein (CRCT1). CRCT1 is part of the human epidermal differentiation complex gene cluster located on chromosome 1q21. This region has been associated with aggressive BC where increases in 1q21.3 copy number (encoding *CRCT1*, *HNRN*, *KPRP*, and *FLG2* genes) result in the repression of CRCT1 in BC. A tumor-suppressive capability for CRCT1 may be linked to its role in promoting cornification, a form of terminal differentiation and apoptosis required for the formation of the outermost skin barrier. In particular, CRCT1 promotes epidermal keratinocytes to undergo cornification.^72^ How far our distinctive observations reflect our demographic group, type of BC, or our focus on urine will be the subject of further studies.

Completely different physiological events could be reported by changes in beta-glucuronidase isoform 1 precursor (β-GUSB) which was highly expressed in the BC group in comparison to the other groups. β-GUSB expression has been primarily associated with the microbiome, and this is relevant as dysbiosis of gut microbiota has been found to be involved in this often estrogen-driven disease.^73^ This could be directly relevant to BC, as the diversity and abundance of the microbiota producing β-GUSB and/or β-galactosidase differed in blood serum samples of BC patients (*n* = 96) and healthy controls (*n* = 192).^74^ β-GUSB and β-galactosidase producing bacteria were predominant in BC patients and healthy control groups, respectively.^74^ Byrd and colleagues^75^ suggested a positive correlation between *Bacteroides* bacteria in the fecal samples of BC patients (*n* = 379), and for every 1% increase in its relative abundance, there was a statistically significant 5% higher odds of developing BC. It was postulated that these could be linked to β-GUSB enzyme activities that lead to BC.^75^

### 4.2 Urinary Proteins Can Distinguish between Preinvasive and Invasive BC

Our further assessment suggested that urinary levels of calpain-2 and cystatin-C proteins could be used to diagnose between preinvasive and invasive BC. Calpain is an intracellular calcium-dependent cysteine-protease family that ubiquitously expressed in mammalian tissues. Most relevant to this study, calpain-2, a classical calpain, plays a vital role in maintaining mammary gland homeostasis and in the progression of BC via various well-characterized signaling pathways.^76–78^ In line with this, RNAi knockdown of calpain-2 in the AC2M2 mouse mammary carcinoma cell line reduced growth and migration in vitro and also decreased tumor growth in vivo using a mammary fat pad engraftment model. This was associated with changes in the lPP2A-Akt-FoxO-p2 and p27 Kip1 tumor suppressors.^78^ In patients, high calpain-2 expression in both basal-like and triple-negative BC was linked to poor BC survival.^79^

Cystatin-C is the best-characterized type-2 cystatin family and the most important extracellular inhibitor of cathepsin-type proteases. Therefore, cystatin-C plays a significant role in regulating extracellular proteolysis. Intriguingly, elevated cystatin-C levels in tumor tissues correlate with a favorable prognosis for cancer patients, while higher levels in body fluids, and presumably in urine, could be associated with poor prognosis. Depletion of cystatin-C adversely affects tumor cell proliferation, leading to reduced tumor growth in an orthotopic mouse model of BC. This effect is due to the complex interplay between cysteine cathepsin activity, cystatin C, 14-3-3 proteins, and transforming growth factor beta (TGF-β) signaling pathways.^80^ Furthermore, the potential of cystatin-C as the BC monitoring markers at several time-points (at diagnosis, following preoperative chemotherapy, and at relapse) has been observed.^81^

## 5 Conclusion

Our findings revealed differentially expressed urinary proteins which are highly associated with BC. These were fibrinogen gamma chain isoform gamma-B precursor, beta-glucuronidase isoform 1 precursor, alpha-actinin-1 isoform X9, peptidase inhibitor 16 precursor, cysteine-rich C-terminal protein 1 isoform, and guanine nucleotide-binding protein G­(I)/G­(S)/G­(T) subunit beta-1 isoform X1 which could distinguish between BC, BBD, SC, and HC groups (*p* < 0.01). The literature suggested that most of these proteins could be associated with BC development, proliferation, and metastasis. Moreover, we found sufficient involvement with innate immunity response. In terms of BC clinical characteristics, the catalytic subunit of calpain-2 isoform 1 and the protease inhibitor precursor cystatin-C were able to differentiate between preinvasive and invasive types of BC. Future studies should extend this study by increasing the number of samples being assessed, particularly of the different types or subtypes. In these further studies, we will also consider renal function, medication use, or menstrual cycle phase. However, the detection of urinary proteins could suggest the presence of BC, which could facilitate the development of a test which can be performed in primary care to help with their triage of urgent cases to the rapid access breast clinics.

## Supplementary Material





## Data Availability

Proteomics data from LC-MSMS analysis has been deposited to the ProteomeXchange Consortium via the PRIDE partner repository: Proteome data set identifier PXD061502

## Notes

Participant clinical metadata (CSV)
Heatmap analysis visualizing the differentially expressed urinary proteins (*p* < 0.001) between BC, BBD, SC, and HC in every samples; Gene ontology classification showing biological processes, cellular components, and molecular functions of differentially expressed proteins in urine samples of BC, BBD, SC, and HC; posthoc analysis with Fisher’s LSD (*P* = < 0.05) identifying the major sources of variation in the urine proteome distinguishing between BC, BBD, SC, and HC; breast tissue proteins identified in urine in this study, related to other studies; differentially expressed proteins based on pairwise comparisons between BC and BBD, SC, and HC; and statistics of top 10 enriched genes and KEGG pathways related to DEP (PDF)
