# Supplementary material for: The Urinary Proteome Differs with the Presence and Type of Breast Cancer
Source: J Proteome Res. 2025 Nov 3;24(12):5932–47. doi: 10.1021/acs.jproteome.5c00229 (PMC12687361; doi:10.1021/acs.jproteome.5c00229)
Supplement: Supplementary file 2 [file pr5c00229_si_002.pdf]

Supporting informtion

The urinary proteome differs with the presence and type of breast cancer

Nur Aimi Aliah Zainurin[1], Russell M. Morphew[1], Alekhya Ganti[1], Dimitra Ivanova[1], Tim Gate[3], Helen Tench[4], Helen Phillips[1], Mandana Pennick[2], Luis A. J. Mur[1*]

[1]Department of Life Sciences, Aberystwyth University, Aberystwyth, SY23 3DA, United Kingdom

[2]Glan Clwyd Hospital, Betsi Cadwaladr University Health Board, Bodelwyddan, LL18 5UJ, United Kingdom

[3]Wrexham Maelor Hospital, Betsi Cadwaladr University Health Board, Wrexham, LL13 7TD, United Kingdom

[4]Bronglais General Hospital, Hywel Dda University Health Board, Aberystwyth, SY23 1ER, United Kingdom

[*]Communicating author; lum@aber.ac.uk

Supporting Information: Table of Contents

Figure S1. Heatmap analysis visualising the differentially expressed urinary proteins ($p < 0.001$) between breast cancer (BC), benign breast disease (BBD), symptom control (SC) and healthy control (HC) in every samples. The hierarchical clustering analysis applied Euclidean distance measure and Ward's clustering method.

Figure S2. Gene ontology classification showing (a) biological processes, (b) cellular components and (c) molecular functions of differentially expressed proteins in urine samples of Breast Cancer (BC), Benign Breast Disease (BBD), Symptom controls (SC) and Healthy control (HC).

Table S1. Participant clinical metadata

Table S2. *Post hoc* analysis with Fisher's LSD (P= < 0.05) identifying the major sources of variation in the urine proteome distinguishing between breast cancer (BC), Benign breast disease (BBD), symptom controls (SC) and healthy control (HC).

Table S3. Breast tissue proteins identified in urine in this study, related to other studies

Table S4. Differentially expressed proteins based on pairwise comparisons between breast cancer (BC) and Benign breast disease (BBD), symptom controls (SC) and healthy control (HC). Fold-change (FC) threshold 2 and false-discovery rate 0.05.

Table S5. Statistics of top 10 enriched gene and KEGG pathways related to DEP.

Figure S1. Heatmap analysis visualising the differentially expressed urinary proteins ($p < 0.001$) between breast cancer (BC), benign breast disease (BBD), symptom control (SC) and healthy control (HC) in every samples. The hierarchical clustering analysis applied Euclidean distance measure and Ward's clustering method.

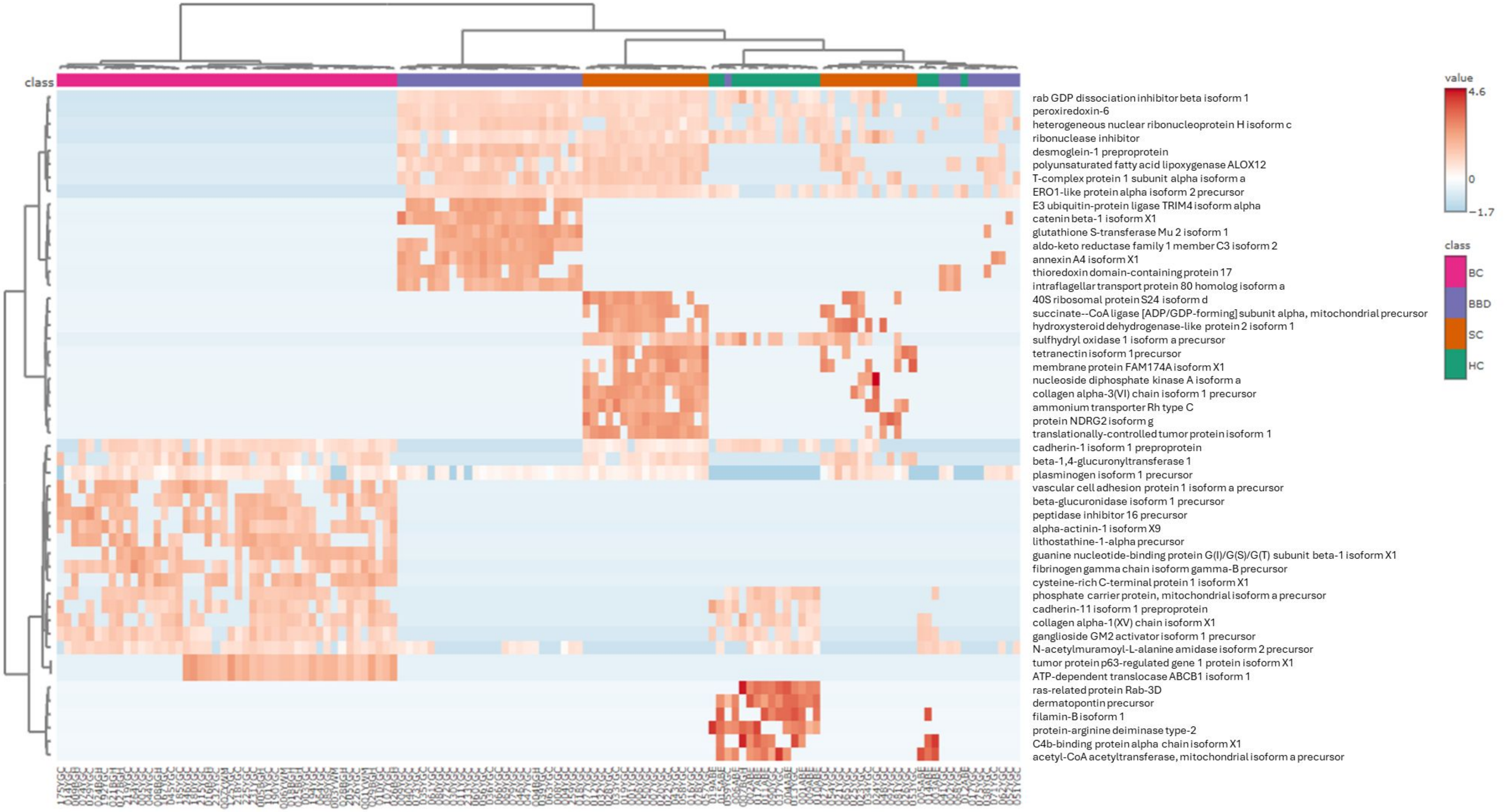

Figure S2. Gene ontology classification showing (a) biological processes, (b) cellular components and (c) molecular functions of differentially expressed proteins in urine samples of breast cancer (BC), benign breast disease (BBD), symptom control (SC) and healthy control (HC).

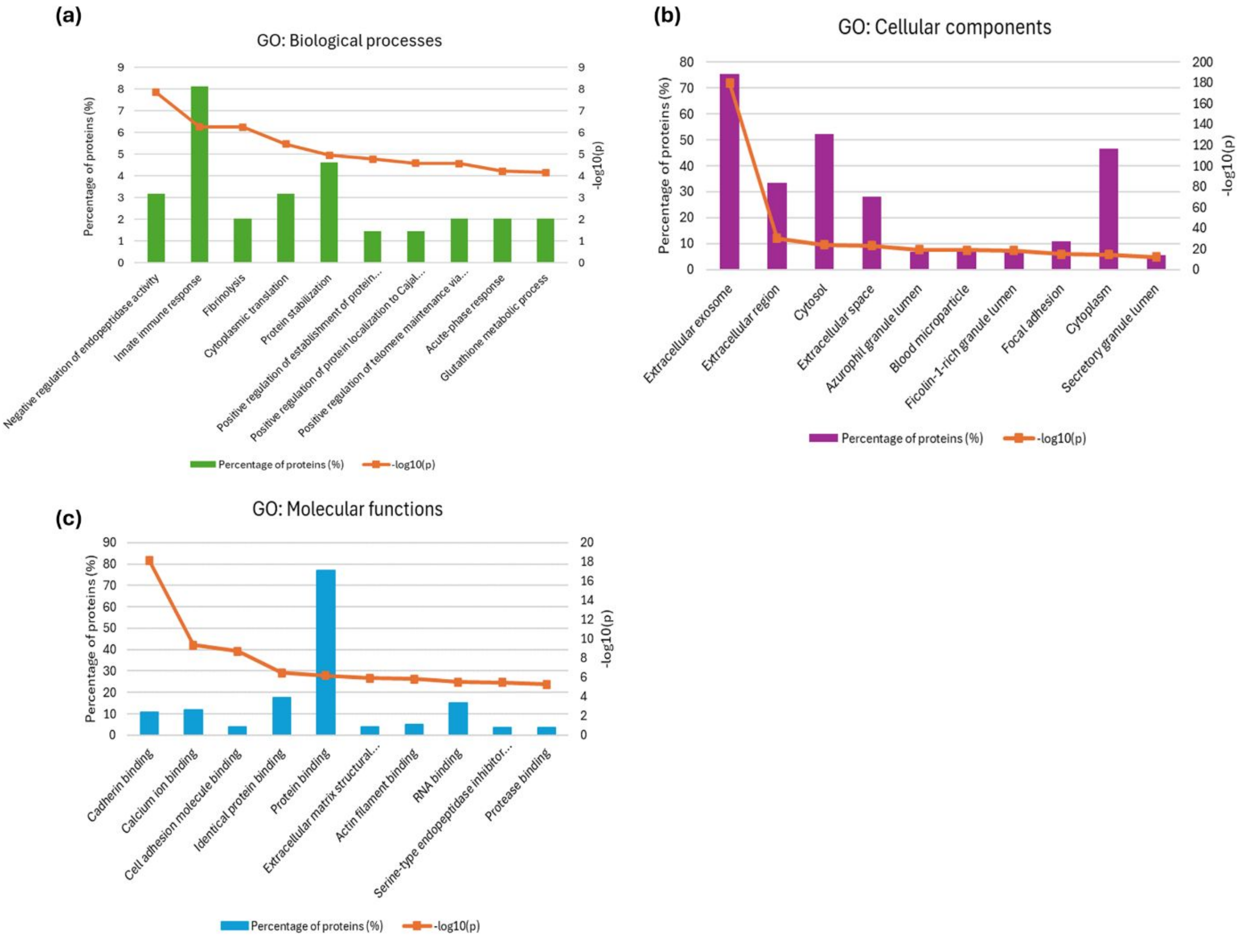

Supplementary Tables

Table S1. Participant clinical metadata (available as a .csv file)

Table S2. *Post hoc* analysis with Fisher's LSD (P= < 0.05) identifying the major sources of variation in the urine proteome distinguishing between breast cancer (BC), Benign breast disease (BBD), symptom controls (SC) and healthy control (HC).

Accession ID	Protein description	f.value	p-value	-log10(p-value)	FDR
NP_068656.2	fibrinogen gamma chain isoform gamma-B precursor	91.156	2.0137e-31	30.696	1.0874e-28
NP_000396.2	ganglioside GM2 activator isoform 1 precursor	86.193	2.1852e-30	29.66	5.9002e-28
NP_001928.2	dermatopontin precursor	63.939	3.4157e-25	24.467	6.1482e-23
NP_000172.2	beta-glucuronidase isoform 1 precursor	56.775	2.7467e-23	22.561	3.7081e-21
XP_011535567.1	alpha-actinin-1 isoform X9	56.222	3.9055e-23	22.408	4.2179e-21
NP_000688.2	polyunsaturated fatty acid lipoxygenase ALOX12	55.331	6.9087e-23	22.161	6.2178e-21
NP_004896.1	peroxiredoxin-6	54.597	1.1098e-22	21.955	8.5614e-21
NP_055399.1	ERO1-like protein alpha isoform 2 precursor	53.663	2.0396e-22	21.69	1.3767e-20
NP_065851.1	intraflagellar transport protein 80 homolog isoform a	52.442	4.5612e-22	21.341	2.7367e-20
NP_001240837.1	aldo-keto reductase family 1 member C3 isoform 2	51.667	7.6368e-22	21.117	4.1239e-20
NP_001273201.1	translationally-controlled tumor protein isoform 1	50.457	1.7237e-21	20.764	8.462e-20
NP_116120.1	thioredoxin domain-containing protein 17	47.528	1.2927e-20	19.889	5.817e-19
XP_016861227.1	catenin beta-1 isoform X1	46.125	3.4736e-20	19.459	1.4429e-18
NP_004360.2	collagen alpha-3(VI) chain isoform 1 precursor	45.551	5.2274e-20	19.282	2.0163e-18
NP_001350475.1	N-acetylmuramoyl-L-alanine amidase isoform 2 precursor	44.161	1.4218e-19	18.847	5.105e-18
NP_001186088.1	peptidase inhibitor 16 precursor	44.076	1.5126e-19	18.82	5.105e-18
NP_002817.2	sulfhydryl oxidase 1 isoform a precursor	43.863	1.7663e-19	18.753	5.6105e-18
NP_003840.2	succinate--CoA ligase [ADP/GDP-forming] subunit alpha, mitochondrial precursor	43.747	1.9217e-19	18.716	5.7388e-18

NP_006867.1	beta-1,4-glucuronyltransferase 1	43.679	2.0192e-19	18.695	5.7388e-18
XP_011507958.1	cysteine-rich C-terminal protein 1 isoform X1	43.231	2.8032e-19	18.552	7.3341e-18
XP_006714663.1	membrane protein FAM174A isoform X1	43.207	2.8521e-19	18.545	7.3341e-18
NP_004274.1	ras-related protein Rab-3D	43.016	3.2831e-19	18.484	8.0586e-18
NP_004351.1	cadherin-1 isoform 1 preproprotein	42.854	3.699e-19	18.432	8.6846e-18
NP_976319.1	ribonuclease inhibitor	42.687	4.1838e-19	18.378	9.4135e-18
XP_047274005.1	guanine nucleotide-binding protein G(I)/G(S)/G(T) subunit beta-1 isoform X1	42.331	5.444e-19	18.264	1.1759e-17
NP_001307970.1	ammonium transporter Rh type C	42.17	6.1338e-19	18.212	1.274e-17
NP_110379.2	T-complex protein 1 subunit alpha isoform a	41.621	9.2361e-19	18.035	1.8472e-17
NP_001373606.1	acetyl-CoA acetyltransferase, mitochondrial isoform a precursor	41.313	1.1631e-18	17.934	2.2432e-17
NP_001157789.1	filamin-B isoform 1	41.236	1.2324e-18	17.909	2.2947e-17
NP_001351156.1	heterogeneous nuclear ribonucleoprotein H isoform c	40.854	1.6422e-18	17.785	2.9559e-17
NP_001485.2	rab GDP dissociation inhibitor beta isoform 1	40.526	2.1047e-18	17.677	3.6663e-17
NP_005879.1	phosphate carrier protein, mitochondrial isoform a precursor	40.24	2.6142e-18	17.583	4.4115e-17
XP_005256758.2	alpha-2-antiplasmin isoform X2	39.702	3.94e-18	17.405	6.1803e-17
XP_047300036.1	annexin A4 isoform X1	39.69	3.9759e-18	17.401	6.1803e-17
NP_000292.1	plasminogen isoform 1 precursor	39.681	4.0058e-18	17.397	6.1803e-17
NP_148977.2	E3 ubiquitin-protein ligase TRIM4 isoform alpha	39.635	4.1493e-18	17.382	6.224e-17
NP_002900.2	lithostathine-1-alpha precursor	39.579	4.3301e-18	17.364	6.3196e-17
NP_031391.2	protein-arginine deiminase type-2	38.985	6.8362e-18	17.165	9.7146e-17
NP_000839.1	glutathione S-transferase Mu 2 isoform 1	38.943	7.0594e-18	17.151	9.7746e-17
NP_001247435.1	40S ribosomal protein S3 isoform 2	38.713	8.4338e-18	17.074	1.1386e-16
NP_001069.1	vascular cell adhesion protein 1 isoform a precursor	38.49	1.0029e-17	16.999	1.3209e-16
NP_001933.2	desmoglein-1 preproprotein	38.14	1.3166e-17	16.881	1.6928e-16
NP_001135757.1	40S ribosomal protein S24 isoform d	37.705	1.849e-17	16.733	2.322e-16
NP_000692.2	sodium/potassium-transporting ATPase subunit alpha-1 isoform a	37.089	3.0031e-17	16.522	3.5337e-16

NP_001335874.1	ATP-dependent translocase ABCB1 isoform 1	37.086	3.0102e-17	16.521	3.5337e-16
XP_024309245.1	tumor protein p63-regulated gene 1 protein isoform X1	37.086	3.0102e-17	16.521	3.5337e-16
XP_047278716.1	collagen alpha-1(XV) chain isoform X1	36.93	3.4033e-17	16.468	3.9102e-16
NP_003269.2	tetranectin isoform 1precursor	36.882	3.5363e-17	16.451	3.9784e-16
NP_001788.2	cadherin-11 isoform 1 preproprotein	36.521	4.7099e-17	16.327	5.1905e-16
NP_004860.2	vacuolar protein sorting-associated protein 4B	36.17	6.2291e-17	16.206	6.7274e-16
NP_001341487.1	protein NDRG2 isoform g	36.065	6.7769e-17	16.169	7.1756e-16
NP_001011.1	40S ribosomal protein S16 isoform 1	35.551	1.0229e-16	15.99	1.0622e-15
XP_047282644.1	tripartite motif-containing protein 29 isoform X1	34.986	1.6154e-16	15.792	1.6459e-15
NP_001264153.1	transgelin-2 isoform a	34.613	2.1875e-16	15.66	2.1875e-15
NP_066949.2	3-mercaptopyruvate sulfurtransferase isoform 1	34.444	2.5098e-16	15.6	2.4642e-15
NP_114141.2	hemicentin-1 precursor	34.407	2.5868e-16	15.587	2.4686e-15
NP_004868.1	glia maturation factor gamma isoform 1	34.398	2.6057e-16	15.584	2.4686e-15
NP_115679.2	hydroxysteroid dehydrogenase-like protein 2 isoform 1	34.288	2.8524e-16	15.545	2.6557e-15
NP_037511.2	dipeptidyl peptidase 2 preproprotein	34.027	3.5321e-16	15.452	3.2328e-15
XP_005273308.1	C4b-binding protein alpha chain isoform X1	33.862	4.0431e-16	15.393	3.6388e-15
NP_006126.1	F-actin-capping protein subunit alpha-1	33.841	4.1146e-16	15.386	3.6425e-15
XP_047285885.1	protein TESPA1 isoform X1	33.272	6.5847e-16	15.181	5.735e-15
NP_001335270.1	syntenin-1 isoform 7	32.868	9.2078e-16	15.036	7.8924e-15
NP_001007.2	40S ribosomal protein S12	32.609	1.1436e-15	14.942	9.6489e-15
NP_002841.3	receptor-type tyrosine-protein phosphatase S isoform 1 precursor	32.516	1.2352e-15	14.908	1.0262e-14
NP_005132.2	fibrinogen beta chain isoform 1 preproprotein	32.306	1.4735e-15	14.832	1.2056e-14
NP_002943.2	40S ribosomal protein S2	32.262	1.5295e-15	14.815	1.2327e-14
NP_066998.1	hepcidin preproprotein	32.2	1.6102e-15	14.793	1.2787e-14
NP_006400.2	actin-related protein 2/3 complex subunit 1A isoform 1	32.132	1.7061e-15	14.768	1.3352e-14
XP_047304261.1	V-type proton ATPase catalytic subunit A isoform X1	31.983	1.9335e-15	14.714	1.4915e-14
NP_963925.2	protein-glutamine gamma-glutamyltransferase 5 isoform 1	31.837	2.1875e-15	14.66	1.6637e-14

NP_006422.1	T-complex protein 1 subunit beta isoform 1	31.788	2.2798e-15	14.642	1.7099e-14
NP_001284506.1	astrocytic phosphoprotein PEA-15 isoform b	31.615	2.6393e-15	14.579	1.9524e-14
NP_003236.3	protein-glutamine gamma-glutamyltransferase E	31.359	3.2798e-15	14.484	2.3934e-14
NP_057071.2	heme-binding protein 1	31.28	3.508e-15	14.455	2.5258e-14
NP_005414.1	trefoil factor 2 precursor	31.195	3.7713e-15	14.424	2.6796e-14
XP_016873621.1	epidermal growth factor receptor kinase substrate 8-like protein 2 isoform X1	30.938	4.6932e-15	14.329	3.2914e-14
NP_536859.1	tyrosine-protein phosphatase non-receptor type 6 isoform 3	30.842	5.0959e-15	14.293	3.5005e-14
NP_003567.2	serine/threonine-protein kinase 24 isoform a precursor	30.836	5.1211e-15	14.291	3.5005e-14
XP_016863675.2	hairy and enhancer of split-related protein HELT isoform X1	30.768	5.4284e-15	14.265	3.6642e-14
NP_001753.1	T-complex protein 1 subunit zeta isoform a	30.7	5.7551e-15	14.24	3.8005e-14
NP_005073.2	E3 ubiquitin/ISG15 ligase TRIM25	30.697	5.7711e-15	14.239	3.8005e-14
NP_938149.2	neutral alpha-glucosidase AB isoform 3 precursor	30.659	5.9633e-15	14.225	3.8798e-14
XP_011508218.1	spectrin alpha chain, erythrocytic 1 isoform X1	30.063	9.966e-15	14.001	6.4067e-14
NP_001338102.1	cytosolic purine 5'-nucleotidase isoform 2	29.525	1.5887e-14	13.799	1.0093e-13
XP_047299182.1	putative V-set and immunoglobulin domain-containing-like protein IGHV4OR15-8	29.493	1.6342e-14	13.787	1.0261e-13
NP_054752.3	methyltransferase-like protein 7A precursor	29.412	1.7545e-14	13.756	1.089e-13
XP_016869381.2	epiplakin isoform X4	29.344	1.861e-14	13.73	1.1419e-13
NP_001078927.1	catenin delta-1 isoform 1ABC	29.302	1.9309e-14	13.714	1.1597e-13
NP_647537.1	attractin isoform 1 preproprotein	29.301	1.9329e-14	13.714	1.1597e-13
NP_001073592.1	major prion protein preproprotein Prp precursor	29.276	1.9756e-14	13.704	1.1723e-13
NP_005175.2	calmodulin-3 isoform 1	29.231	2.0544e-14	13.687	1.2058e-13
NP_937818.1	nucleoside diphosphate kinase A isoform a	28.989	2.5399e-14	13.595	1.4748e-13
NP_001092315.2	annexin A8-like protein 1 isoform 1	28.918	2.7036e-14	13.568	1.5531e-13
XP_047287783.1	corticosteroid-binding globulin isoform X1	28.892	2.7661e-14	13.558	1.5574e-13
NP_036205.1	T-complex protein 1 subunit epsilon isoform a	28.891	2.7687e-14	13.558	1.5574e-13
XP_005248694.1	heterogeneous nuclear ribonucleoprotein Q isoform X1	28.846	2.8781e-14	13.541	1.6023e-13

NP_001275905.1	X-ray repair cross-complementing protein 6 isoform 1	28.669	3.3637e-14	13.473	1.8535e-13
NP_001036189.1	nectin-2 isoform delta precursor	28.626	3.4941e-14	13.457	1.9059e-13
NP_000261.2	purine nucleoside phosphorylase	28.506	3.8851e-14	13.411	2.098e-13
NP_004628.4	ras-related protein Rab-7a	28.448	4.0891e-14	13.388	2.1736e-13
NP_061036.3	ubiquitin thioesterase OTU1 isoform 1	28.442	4.1105e-14	13.386	2.1736e-13
XP_011507882.1	neutrophil cytosol factor 2 isoform X1	28.433	4.1459e-14	13.382	2.1736e-13
NP_001716.3	bactericidal permeability-increasing protein precursor	28.397	4.2779e-14	13.369	2.2212e-13
NP_858060.2	serine/threonine-protein phosphatase 2A 55 kDa regulatory subunit B beta isoform isoform a	28.229	4.9629e-14	13.304	2.5524e-13
XP_047302795.1	BRO1 domain-containing protein BROX isoform X1	27.797	7.2893e-14	13.137	3.7134e-13
NP_004083.3	enoyl-CoA hydratase, mitochondrial	27.663	8.2192e-14	13.085	4.1155e-13
NP_064695.3	aldo-keto reductase family 1 member B10	27.652	8.3007e-14	13.081	4.1155e-13
NP_001291217.1	40S ribosomal protein SA isoform 2	27.651	8.3073e-14	13.081	4.1155e-13
NP_000840.2	glutathione S-transferase Mu 3	27.538	9.1906e-14	13.037	4.5117e-13
NP_001257293.1	cytosolic 10-formyltetrahydrofolate dehydrogenase isoform 1	27.244	1.1965e-13	12.922	5.8208e-13
NP_001702.1	biglycan preproprotein	27.215	1.2279e-13	12.911	5.9202e-13
NP_000388.2	cytochrome b-245 heavy chain	27.066	1.4047e-13	12.852	6.7128e-13
NP_002954.2	protein S100-A7	26.942	1.5707e-13	12.804	7.4403e-13
NP_997647.2	fibronectin isoform 1 precursor	26.757	1.857e-13	12.731	8.7197e-13
NP_003093.2	extracellular superoxide dismutase [Cu-Zn] preproprotein	26.621	2.0993e-13	12.678	9.7727e-13
NP_001333790.1	tuftelin-interacting protein 11 isoform 1	26.596	2.1483e-13	12.668	9.9153e-13
NP_006057.1	aldo-keto reductase family 1 member A1	26.585	2.1695e-13	12.664	9.9283e-13
NP_000681.2	aldehyde dehydrogenase, mitochondrial isoform 1 precursor	26.54	2.2593e-13	12.646	1.0252e-12
NP_006420.1	T-complex protein 1 subunit eta isoform a	26.342	2.706e-13	12.568	1.2177e-12
NP_000748.4	macrophage colony-stimulating factor 1 isoform a precursor	26.298	2.8176e-13	12.55	1.2574e-12

NP_004354.3	carcinoembryonic antigen-related cell adhesion molecule 5 isoform 1 preproprotein	26.197	3.088e-13	12.51	1.3668e-12
NP_001020.2	40S ribosomal protein S26	26.163	3.1849e-13	12.497	1.3983e-12
NP_006547.1	phosphomevalonate kinase isoform 1	26.123	3.306e-13	12.481	1.4354e-12
NP_002768.3	myeloblastin precursor	26.117	3.3228e-13	12.479	1.4354e-12
NP_002067.1	N-acetylglucosamine-6-sulfatase precursor	26.06	3.5003e-13	12.456	1.5001e-12
NP_000080.2	collagen alpha-2(I) chain precursor	25.991	3.7297e-13	12.428	1.5858e-12
NP_000851.2	15-hydroxyprostaglandin dehydrogenase [NAD(+)] isoform 1	25.888	4.0983e-13	12.387	1.729e-12
NP_116171.3	hepatitis A virus cellular receptor 2 precursor	25.722	4.7722e-13	12.321	1.9977e-12
NP_000190.1	N-sulphoglucosamine sulphohydrolase isoform 1 precursor	25.707	4.837e-13	12.315	2.0092e-12
NP_003739.2	delta-1-pyrroline-5-carboxylate dehydrogenase, mitochondrial isoform a precursor	25.479	5.9696e-13	12.224	2.4607e-12
XP_005247472.1	histidine-rich glycoprotein isoform X1	25.404	6.393e-13	12.194	2.6153e-12
NP_036519.2	protein-arginine deiminase type-4	25.312	6.9589e-13	12.157	2.8254e-12
NP_001362207.1	complement factor I isoform 4 preproprotein	25.259	7.3132e-13	12.136	2.9471e-12
NP_000966.2	60S ribosomal protein L11 isoform 1	25.092	8.533e-13	12.069	3.4132e-12
NP_000476.1	adenine phosphoribosyltransferase isoform a	24.969	9.5622e-13	12.019	3.7968e-12
XP_047277377.1	elongation factor 1-delta isoform X1	24.95	9.7374e-13	12.012	3.8381e-12
NP_001361773.1	protein NDRG1 isoform 4	24.905	1.0153e-12	11.993	3.9731e-12
NP_002566.1	plasminogen activator inhibitor 2	24.865	1.0536e-12	11.977	4.093e-12
XP_011541721.1	peptidyl-glycine alpha-amidating monooxygenase isoform X1	24.791	1.1284e-12	11.948	4.3525e-12
NP_006089.1	receptor of activated protein C kinase 1	24.772	1.1485e-12	11.94	4.3984e-12
NP_000599.1	alpha-1-acid glycoprotein 2 precursor	24.757	1.1655e-12	11.933	4.4323e-12
NP_446464.1	argininosuccinate synthase	24.712	1.2152e-12	11.915	4.5887e-12
XP_011511158.1	neprilysin isoform X1	24.659	1.276e-12	11.894	4.7848e-12
NP_001168.1	ADP-ribosylation factor-like protein 1 isoform 1	24.397	1.63e-12	11.788	6.0703e-12
XP_005244806.1	agrin isoform X1	24.34	1.7203e-12	11.764	6.3629e-12

NP_000419.1	latent-transforming growth factor beta-binding protein 2 precursor	24.164	2.0282e-12	11.693	7.4505e-12
NP_001265126.1	CDK5 regulatory subunit-associated protein 3 isoform c	24.152	2.0525e-12	11.688	7.4889e-12
NP_733746.1	sialate O-acetylesterase isoform 1 precursor	24.045	2.2691e-12	11.644	8.2234e-12
XP_047303524.1	dystroglycan 1 isoform X1	23.965	2.4466e-12	11.611	8.8076e-12
XP_047298127.1	plastin-3 isoform X1	23.535	3.6746e-12	11.435	1.3141e-11
NP_000959.2	60S ribosomal protein L4	23.457	3.9544e-12	11.403	1.4048e-11
NP_001258867.1	multiple epidermal growth factor-like domains protein 8 isoform 1 precursor	23.278	4.6902e-12	11.329	1.6554e-11
NP_006699.2	lactoylglutathione lyase	23.193	5.0862e-12	11.294	1.7835e-11
NP_000628.2	glutathione reductase, mitochondrial isoform 1 precursor	23.083	5.6493e-12	11.248	1.9681e-11
XP_047274226.1	beta/gamma crystallin domain-containing protein 1 isoform X1	23.025	5.9697e-12	11.224	2.0664e-11
XP_047276968.1	maltase-glucoamylase isoform X1	22.84	7.126e-12	11.147	2.451e-11
NP_001634.1	apolipoprotein A-II preproprotein	22.762	7.6818e-12	11.115	2.6254e-11
NP_065392.1	adipocyte plasma membrane-associated protein	22.68	8.3092e-12	11.08	2.822e-11
NP_001015878.1	aurora kinase C isoform 1	22.623	8.7787e-12	11.057	2.9628e-11
XP_011525231.1	suprabasin isoform X1	22.257	1.2491e-11	10.903	4.1848e-11
NP_000033.2	beta-2-glycoprotein 1 precursor	22.251	1.2555e-11	10.901	4.1848e-11
XP_024306539.1	alpha-N-acetylglucosaminidase isoform X1	21.975	1.6404e-11	10.785	5.4344e-11
NP_001308349.1	rho GDP-dissociation inhibitor 2	21.905	1.7554e-11	10.756	5.7801e-11
XP_047293580.1	cytosolic non-specific dipeptidase isoform X1	21.104	3.8429e-11	10.415	1.2577e-10
NP_055439.1	protein S100-A6	21.074	3.9591e-11	10.402	1.2879e-10
NP_060676.2	vacuolar protein sorting-associated protein 35	21.03	4.1312e-11	10.384	1.3358e-10
NP_000473.2	apolipoprotein A-IV precursor	21.018	4.1806e-11	10.379	1.3438e-10
NP_000330.3	solute carrier family 12 member 3 isoform 1	20.79	5.2339e-11	10.281	1.6724e-10
NP_004306.3	acid ceramidase isoform b	20.767	5.3583e-11	10.271	1.702e-10
NP_061882.2	toll-interacting protein isoform 1	20.709	5.6726e-11	10.246	1.7913e-10
NP_001303260.1	ABHD14A-ACY1 readthrough (NMD candidate)	20.603	6.3004e-11	10.201	1.978e-10
NP_001289978.1	myosin regulatory light chain 12A isoform 2	20.556	6.6009e-11	10.18	2.0604e-10

NP_005969.2	protein S100-A2 isoform 1	20.435	7.444e-11	10.128	2.3102e-10
NP_001728.1	complement component C9 preproprotein	20.372	7.9266e-11	10.101	2.4459e-10
NP_065137.1	endosialin precursor	20.29	8.5993e-11	10.066	2.6384e-10
NP_001146.2	annexin A6 isoform 1	20.272	8.7536e-11	10.058	2.6556e-10
NP_002449.2	mucin-5B precursor	20.272	8.7536e-11	10.058	2.6556e-10
NP_001963.1	neutrophil elastase preproprotein	20.195	9.4537e-11	10.024	2.852e-10
NP_006861.1	destrin isoform a	20.1	1.0395e-10	9.9832	3.1184e-10
NP_055040.2	serine/threonine-protein phosphatase 2A 65 kDa regulatory subunit A alpha isoform isoform 1	19.922	1.2412e-10	9.9062	3.7031e-10
NP_443204.1	leucine-rich alpha-2-glycoprotein precursor	19.446	2.0022e-10	9.6985	5.9407e-10
NP_115914.1	tubulin beta-6 chain isoform 1	19.4	2.0974e-10	9.6783	6.189e-10
NP_002776.3	pregnancy-specific beta-1-glycoprotein 11 isoform 1 precursor	19.04	3.0198e-10	9.52	8.8625e-10
NP_057630.2	complement C1r subcomponent-like protein isoform 1 precursor	18.889	3.5212e-10	9.4533	1.0278e-09
NP_001888.2	chondroitin sulfate proteoglycan 4 precursor	18.732	4.132e-10	9.3838	1.1996e-09
NP_001374479.1	serine/threonine-protein kinase MRCK alpha isoform F	18.623	4.6194e-10	9.3354	1.3339e-09
XP_011512750.1	N(G),N(G)-dimethylarginine dimethylaminohydrolase 2 isoform X1	18.431	5.6215e-10	9.2501	1.6147e-09
NP_001020402.1	keratinocyte proline-rich protein	18.408	5.7566e-10	9.2398	1.6447e-09
NP_001090.2	prostatic acid phosphatase isoform PAP precursor	18.154	7.4676e-10	9.1268	2.1224e-09
NP_001289855.1	bone marrow proteoglycan isoform 1 preproprotein	18.066	8.1763e-10	9.0874	2.3116e-09
XP_006714459.1	general vesicular transport factor p115 isoform X1	18.045	8.3564e-10	9.078	2.3502e-09
NP_066952.1	inorganic pyrophosphatase	18.014	8.6243e-10	9.0643	2.413e-09
NP_057936.1	folate receptor alpha precursor	17.889	9.8118e-10	9.0083	2.7311e-09
NP_001035207.1	arf-GAP with Rho-GAP domain, ANK repeat and PH domain-containing protein 1 isoform c	17.817	1.0578e-09	8.9756	2.9192e-09
NP_055851.1	endonuclease domain-containing 1 protein precursor	17.815	1.0596e-09	8.9749	2.9192e-09
NP_059447.2	major vault protein isoform 1	17.773	1.1065e-09	8.9561	3.0329e-09
NP_001358649.1	mucin-1 isoform 22 precursor	17.711	1.1797e-09	8.9282	3.2173e-09

NP_000393.4	glucose-6-phosphate 1-dehydrogenase isoform a	17.309	1.7921e-09	8.7466	4.863e-09
NP_001188309.2	contactin-associated protein-like 3B precursor	16.887	2.788e-09	8.5547	7.5276e-09
NP_001334883.1	rabphilin-3A isoform 1	16.648	3.5839e-09	8.4456	9.6284e-09
NP_001289617.1	apolipoprotein E isoform a precursor	16.525	4.0785e-09	8.3895	1.0903e-08
NP_112420.1	heterogeneous nuclear ribonucleoprotein A1 isoform b	16.258	5.4108e-09	8.2667	1.4367e-08
NP_005808.3	perilipin-3 isoform 1	16.255	5.4274e-09	8.2654	1.4367e-08
NP_005313.1	histone H1.5	16.123	6.2484e-09	8.2042	1.6459e-08
NP_570602.2	alpha-1B-glycoprotein precursor	16.054	6.7202e-09	8.1726	1.7616e-08
NP_001186040.1	malate dehydrogenase, cytoplasmic isoform 2	15.992	7.1788e-09	8.1439	1.8727e-08
NP_001316801.1	aquaporin-1 isoform 5	15.925	7.7117e-09	8.1128	2.0021e-08
NP_001139281.1	myosin-14 isoform 3	15.794	8.8666e-09	8.0522	2.2909e-08
NP_006746.1	transaldolase	15.779	9.0099e-09	8.0453	2.3168e-08
NP_001191236.1	vitamin D-binding protein isoform 3 precursor	15.348	1.431e-08	7.8444	3.6623e-08
NP_001001414.1	F-box only protein 50	15.226	1.631e-08	7.7875	4.1544e-08
NP_054706.1	vinculin isoform meta-VCL	15.204	1.671e-08	7.777	4.2364e-08
XP_047286715.1	stAR-related lipid transfer protein 13 isoform X1	15.153	1.7643e-08	7.7534	4.452e-08
NP_005756.2	renin receptor precursor	15.043	1.9887e-08	7.7014	4.9949e-08
XP_047281210.1	ATP synthase subunit gamma, mitochondrial isoform X1	14.827	2.5131e-08	7.5998	6.2829e-08
NP_001317277.1	TBC1 domain family member 8 isoform 1	14.752	2.7264e-08	7.5644	6.7847e-08
NP_008876.3	small proline-rich protein 2D	13.78	7.8984e-08	7.1025	1.9565e-07
NP_009057.1	transitional endoplasmic reticulum ATPase isoform 1	13.621	9.4216e-08	7.0259	2.3231e-07
NP_001304264.1	complement factor D isoform 2 precursor	13.502	1.0739e-07	6.969	2.636e-07
XP_016885317.1	zinc finger protein 185 isoform X7	13.455	1.1315e-07	6.9463	2.7649e-07
NP_000382.3	tripeptidyl-peptidase 1 preproprotein	13.204	1.4961e-07	6.825	3.6392e-07
NP_004153.2	ras-related protein Rab-5A isoform 1	13.04	1.7967e-07	6.7455	4.3509e-07
NP_002078.1	progranulin precursor	13.009	1.8605e-07	6.7304	4.4852e-07
NP_653247.1	immunoglobulin J chain precursor	11.703	8.1393e-07	6.0894	1.9534e-06
NP_001950.1	elongation factor 1-beta	11.672	8.4241e-07	6.0745	2.0128e-06
NP_776213.1	interleukin-1 receptor antagonist protein isoform 2	11.599	9.1581e-07	6.0382	2.1786e-06
NP_036226.2	peroxiredoxin-5, mitochondrial isoform L precursor	11.422	1.1221e-06	5.95	2.6576e-06

XP_047279332.1	neutrophil gelatinase-associated lipocalin isoform X1	11.376	1.183e-06	5.927	2.7895e-06
NP_004660.2	chloride intracellular channel protein 3	11.101	1.6228e-06	5.7897	3.81e-06
NP_001395.1	elongation factor 1-gamma	10.96	1.9097e-06	5.719	4.4642e-06
XP_005259353.1	calpain small subunit 1 isoform X1	10.932	1.9739e-06	5.7047	4.5945e-06
NP_001176.1	zinc-alpha-2-glycoprotein precursor	10.595	2.9151e-06	5.5353	6.756e-06
NP_000290.2	plakophilin-1 isoform 1b	10.499	3.2617e-06	5.4866	7.527e-06
XP_011539528.1	D-3-phosphoglycerate dehydrogenase isoform X1	10.229	4.4675e-06	5.3499	1.0266e-05
XP_047291290.1	glutathione hydrolase 6 isoform X2	10.121	5.0722e-06	5.2948	1.1606e-05
NP_068831.1	junction plakoglobin	9.9176	6.4395e-06	5.1912	1.4672e-05
NP_001303969.2	beta-galactosidase isoform d precursor	9.8734	6.7831e-06	5.1686	1.539e-05
NP_057370.1	L-xylulose reductase isoform 1	9.8117	7.2939e-06	5.137	1.648e-05
NP_000598.2	alpha-1-acid glycoprotein 1 precursor	9.7073	8.2491e-06	5.0836	1.856e-05
NP_937863.1	U2 small nuclear ribonucleoprotein B"	9.6465	8.8625e-06	5.0524	1.9858e-05
NP_001347.3	ATP-dependent RNA helicase DDX3X isoform 1	9.6117	9.2351e-06	5.0346	2.0607e-05
NP_055945.2	TBC1 domain family member 9	9.5038	1.0492e-05	4.9792	2.3315e-05
NP_001300870.1	transforming protein RhoA isoform 1 precursor	9.235	1.4433e-05	4.8407	3.1941e-05
NP_940862.2	guanylate-binding protein 6 isoform 1	8.9423	2.0455e-05	4.6892	4.5084e-05
NP_001288172.1	rho GDP-dissociation inhibitor 1 isoform e	8.8214	2.3635e-05	4.6265	5.1881e-05
NP_002005.1	peptidyl-prolyl cis-trans isomerase FKBP4	8.7256	2.6507e-05	4.5766	5.795e-05
NP_005712.1	actin-related protein 3 isoform 1	8.715	2.6846e-05	4.5711	5.8456e-05
NP_060037.4	N-acetyl-D-glucosamine kinase isoform 1	8.5374	3.3225e-05	4.4785	7.2053e-05
NP_002696.4	periplakin	8.4436	3.7194e-05	4.4295	8.0146e-05
NP_000055.2	complement C3 preproprotein	8.4423	3.7253e-05	4.4288	8.0146e-05
NP_001926.2	dipeptidyl peptidase 4 isoform 1	8.0664	5.8645e-05	4.2318	0.00012567
NP_001186797.1	glutaredoxin-3 isoform 1	7.9717	6.5781e-05	4.1819	0.0001404
NP_000627.2	superoxide dismutase [Mn], mitochondrial isoform A precursor	7.9681	6.6064e-05	4.18	0.00014045
NP_001869.1	cellular retinoic acid-binding protein 2	7.9614	6.6606e-05	4.1765	0.00014105

NP_001278917.1	carboxypeptidase N subunit 2 precursor	7.8567	7.5629e-05	4.1213	0.00015953
NP_001368852.1	aminopeptidase N	7.8369	7.7478e-05	4.1108	0.00016279
NP_597677.2	nipped-B-like protein isoform A	7.7929	8.1725e-05	4.0876	0.00017105
NP_005264.2	guanine nucleotide-binding protein G(I)/G(S)/G(T) subunit beta-2	7.7705	8.3983e-05	4.0758	0.0001751
XP_005259077.1	epidermal growth factor receptor kinase substrate 8-like protein 1 isoform X1	7.7356	8.7633e-05	4.0573	0.00018201
NP_006133.1	14-3-3 protein sigma	7.6878	9.2877e-05	4.0321	0.00019216
NP_005176.1	calmodulin-like protein 3	7.6582	9.629e-05	4.0164	0.00019846
NP_000993.1	60S acidic ribosomal protein P0	7.5825	0.0001056	3.9763	0.00021682
NP_003869.1	gamma-glutamyl hydrolase precursor	7.5476	0.00011019	3.9578	0.0002254
NP_001092304.1	homeobox protein GBX-1	7.4101	0.00013036	3.8849	0.00026563
NP_001121134.1	gelsolin isoform b	7.3489	0.00014048	3.8524	0.00028519
NP_996554.2	osteoclast-associated immunoglobulin-like receptor isoform 1 precursor	7.3184	0.00014583	3.8361	0.00029494
XP_006718761.1	calpain-1 catalytic subunit isoform X1	7.3004	0.00014908	3.8266	0.00030038
NP_005909.2	malate dehydrogenase, mitochondrial isoform 1 precursor	7.0216	0.00020993	3.6779	0.00042142
NP_000945.3	prostaglandin-H2 D-isomerase precursor	6.8199	0.00026913	3.57	0.00053825
NP_000933.1	peptidyl-prolyl cis-trans isomerase B precursor	6.7918	0.00027863	3.555	0.0005552
XP_005249786.1	heterogeneous nuclear ribonucleoproteins A2/B1 isoform X1	6.693	0.00031477	3.502	0.00062492
NP_000843.1	glutathione S-transferase P	6.6039	0.00035144	3.4541	0.00069516
NP_001017963.2	heat shock protein HSP 90-alpha isoform 1	6.5206	0.00038961	3.4094	0.00076784
NP_000745.1	catechol O-methyltransferase isoform MB-COMT	6.5046	0.00039742	3.4007	0.0007804
NP_001193997.1	eIF5-mimic protein 2 isoform 2	6.3612	0.00047479	3.3235	0.00092893
NP_079032.2	multimerin-2 precursor	6.2769	0.00052723	3.278	0.0010278
NP_001748.1	carbonyl reductase [NADPH] 1 isoform 1	6.236	0.0005547	3.2559	0.0010775
NP_001294852.1	serpin B13 isoform 1	6.2309	0.00055826	3.2532	0.0010805
XP_016873759.1	neuroblast differentiation-associated protein AHNAK isoform X2	6.1411	0.00062428	3.2046	0.001204
NP_002298.1	galectin-7	6.0077	0.00073715	3.1324	0.0014166
XP_047292343.1	proline-rich protein 11 isoform X1	5.9864	0.00075699	3.1209	0.0014496

NP_008850.1	serpin B3	5.9427	0.00079943	3.0972	0.0015254
NP_001289958.1	plakophilin-3 isoform PKP3b	5.7732	0.00098805	3.0052	0.0018787
NP_004516.2	low-density lipoprotein receptor-related protein 2 precursor	5.7677	0.00099485	3.0022	0.001885
NP_060697.4	ubiquitin-like modifier-activating enzyme 6	5.7603	0.0010041	2.9982	0.0018958
XP_005253288.1	CD44 antigen isoform X1	5.4698	0.0014452	2.8401	0.0027193
NP_005338.1	endoplasmic reticulum chaperone BiP precursor	5.3937	0.0015902	2.7985	0.0029816
NP_001155901.1	programmed cell death 6-interacting protein isoform 2	5.3795	0.0016187	2.7908	0.0030246
XP_011540530.1	F-actin-capping protein subunit beta isoform X1	5.3748	0.0016285	2.7882	0.0030324
NP_001035110.1	monocyte differentiation antigen CD14 precursor	5.3327	0.0017168	2.7653	0.0031859
XP_005267043.1	mannosyl-oligosaccharide 1,2-alpha-mannosidase IA isoform X1	5.3237	0.0017364	2.7603	0.0032112
NP_001624.1	protein AMBP preproprotein	5.2895	0.0018128	2.7416	0.003341
NP_733821.1	lamin isoform A	5.282	0.0018299	2.7376	0.003361
NP_001171597.1	immunoglobulin lambda-like polypeptide 5 isoform 1	5.2543	0.0018951	2.7224	0.0034689
NP_001899.1	cathepsin B isoform 1 preproprotein	5.1507	0.0021591	2.6657	0.0039389
NP_005558.1	galectin-3-binding protein precursor	5.0346	0.0024994	2.6022	0.0045443
NP_001104547.1	ezrin	4.9892	0.0026468	2.5773	0.0047962
NP_001276675.1	glyceraldehyde-3-phosphate dehydrogenase isoform 1	4.87	0.0030767	2.5119	0.0055567
NP_000091.1	cystatin-B	4.8245	0.0032592	2.4869	0.0058665
NP_003861.1	ras GTPase-activating-like protein IQGAP1	4.8182	0.0032853	2.4834	0.0058938
NP_061928.4	roundabout homolog 4 isoform 1 precursor	4.8152	0.0032974	2.4818	0.005896
XP_047298376.1	ubiquitin-like modifier-activating enzyme 1 isoform X1	4.7413	0.0036205	2.4412	0.0064524
XP_006724746.1	thyroxine-binding globulin isoform X1	4.6179	0.0042331	2.3733	0.0075192
NP_002854.3	glycogen phosphorylase, liver form isoform 1	4.5875	0.0043992	2.3566	0.0077888
NP_001258898.1	heat shock protein HSP 90-beta isoform a	4.5233	0.0047717	2.3213	0.0084207
XP_011533974.1	protein MMS22-like isoform X1	4.5108	0.0048481	2.3144	0.0085277
NP_000202.3	integrin beta-2 isoform 1 precursor	4.4439	0.0052774	2.2776	0.0092526
NP_004406.2	desmoplakin isoform I	4.4133	0.0054864	2.2607	0.0095879
NP_004090.4	stomatin isoform a	4.3177	0.0061938	2.208	0.010789

NP_002995.1	secreted and transmembrane protein 1 precursor	4.2954	0.0063719	2.1957	0.011064
XP_011510737.1	ceruloplasmin isoform X1	4.2893	0.0064217	2.1924	0.011114
NP_525127.1	protein S100-A16	4.2864	0.0064451	2.1908	0.011119
NP_009224.2	complement C4-A isoform 1 preproprotein	4.2705	0.0065769	2.182	0.011311
XP_011526798.2	endothelial protein C receptor isoform X2	4.2155	0.0070527	2.1516	0.01209
NP_002278.2	leukocyte-associated immunoglobulin-like receptor 1 isoform a precursor	4.2041	0.0071553	2.1454	0.012227
NP_001302466.1	L-lactate dehydrogenase B chain isoform LDHBx	4.2007	0.0071864	2.1435	0.012242
NP_001116847.1	histone H3.2	4.114	0.0080241	2.0956	0.013626
NP_001353064.1	UMP-CMP kinase isoform c	4.0917	0.008255	2.0833	0.013974
NP_000173.2	trifunctional enzyme subunit alpha, mitochondrial precursor	4.052	0.0086825	2.0614	0.014652
NP_001143.2	ADP/ATP translocase 2	3.967	0.0096734	2.0144	0.016273
NP_001028189.2	beta-defensin 135 precursor	3.9384	0.010033	1.9986	0.016825
NP_006409.3	olfactomedin-4 precursor	3.8887	0.010687	1.9711	0.017867
NP_005611.1	protein S100-A11	3.8854	0.010732	1.9693	0.017887
NP_002622.2	6-phosphogluconate dehydrogenase, decarboxylating isoform 1	3.8105	0.011806	1.9279	0.019616
NP_001704.2	betaine--homocysteine S-methyltransferase 1	3.7511	0.012734	1.895	0.021093
XP_011539620.1	basement membrane-specific heparan sulfate proteoglycan core protein isoform X1	3.715	0.013333	1.8751	0.022018
NP_000362.1	transthyretin precursor	3.6876	0.013806	1.8599	0.02273
NP_112576.1	SH3 domain-binding glutamic acid-rich-like protein 3	3.6698	0.014122	1.8501	0.023179
XP_011529261.1	moesin isoform X1	3.5834	0.015766	1.8023	0.025798
NP_066299.2	myosin light polypeptide 6 isoform 1	3.5747	0.015941	1.7975	0.026007
NP_004095.4	fatty acid synthase	3.5603	0.016236	1.7895	0.026407
NP_002630.2	serpin B5	3.5115	0.017277	1.7625	0.028016
NP_006301.3	puromycin-sensitive aminopeptidase isoform 1	3.4663	0.018301	1.7375	0.029552
NP_112243.1	ras-related protein Rab-1B	3.465	0.018333	1.7368	0.029552
NP_005800.3	peroxiredoxin-2	3.4575	0.018507	1.7327	0.029744
NP_002965.1	serpin B4 isoform 1	3.4321	0.019117	1.7186	0.030633
NP_006807.1	vesicular integral-membrane protein VIP36 precursor	3.3703	0.020683	1.6844	0.033043
NP_001407.1	eukaryotic initiation factor 4A-I isoform 1	3.3678	0.020748	1.683	0.03305
NP_000909.2	protein disulfide-isomerase precursor	3.364	0.02085	1.6809	0.033114

XP_005252192.3	protein Niban 2 isoform X1	3.3049	0.02248	1.6482	0.035599
NP_001002236.1	alpha-1-antitrypsin precursor	3.2494	0.024127	1.6175	0.038095
NP_000350.1	protein-glutamine gamma-glutamyltransferase K	3.2263	0.024847	1.6047	0.039117
NP_000499.1	fibrinogen alpha chain isoform alpha-E preproprotein	3.2072	0.025461	1.5941	0.039968
NP_001034438.1	EGF-containing fibulin-like extracellular matrix protein 1 precursor	3.2038	0.02557	1.5923	0.040023
NP_000241.1	myeloperoxidase precursor	3.0768	0.030057	1.5221	0.04691

Table S3. Breast tissue proteins identified in urine in this study, related to other studies

Present study						
UniProt	Al-Wajeeh et al. (2020)				Neagu et al. (2022)	Sinha et al. (2023)
DDX3X	A1BG[d]*	CNDP2	HNRNPA2B1	PYGL	COL1A2	• Saliva
CSF1	ACAT1	COL6A3	HRG[d]	RNH1	CTNNB1	CP[d]
PRG2	ACTN1	CP[d]	HSDL2	RPL11	FASN[b]	FN1[d]
MUC1	ACTR3	CRABP2	HSP90AA1	RPL4	S100A6[b]	GSN[d]
DPP4	AHNAK	CTSB	HSP90AB1	RPS12		GSTP1[d]
LRP2	AKR1A1	CYBB	HSPA5	RPS16		IGLL5
PTPRS	AKR1C3	DCXR	HSPG2	RPS2		LGALS3BP[d]
DAG1	ALDH2	DDAH2	IQGAP1	RPS24		MUC5B
NIPBL	AMBP*	DSP	ITGB2	RPS3		NPEPPS [d]
MMRN2	ANPEP*	DSTN	JCHAIN	RPSA		P4HB [d]
CD248	ANXA4	ECHS1	LCN2	S100A11		PGD [d]
FLNB[a]	ANXA6	EEF1B2	LDHB	S100A7		PNP [d]
CD44[a]	APMAP	EEF1D	LGALS3BP[d]	SDCBP		PPIB[d]
PRDX5[a]	APOA2[d]	EEF1G	LMAN2	SERPINA1[d]		PRTN3[d]
RAB7A[a]	APOA4*	EFEMP1	LMNA	SERPINA6[d]		QSOX1
RHOA[a]	APOE	EIF4A1	LRG1[d]	SERPINA7[d]		SERPINB13
RACK1[a]	APOH[d]	ELANE	MDH1	SFN[d]		SFN[d]
	ARHGDIA	ERO1A	MDH2	SLC25A3		STOM[d]
	ARHGDIB	FGA[d]*	MPO	SLC25A5		TALDO1
	ARL1	FGB	MSN	SOD2		TGM3
	ASAH1	FGG	MVP	SOD3		VCP[d]

	ATP1A1	FKBP4	MYL12A	STOM[d]		AZGP1[c,d]
	ATP5F1C	FN1[d]	MYL6	SYNCRIP		PRDX2 [c,d]
	ATP6V1A	G6PD	NAGK	TAGLN2		TTR [c]
	AZGP1[d]	GANAB	NDRG1	TCP1		
	C3[d]*	GAPDH	NPEPPS [d]	TMT1A		• Serum
	C4BPA[d]	GC	ORM1[d]*	TPT1		A1BG[d]
	C9	GDI2	ORM2	TXNDC17		APOA2[d]
	CAPN1	GGH	P4HB [d]	UBA1		APOH[d]
	CAPNS1	GLO1	PDCD6IP	VCL[d]		C3[d]
	CAPZA1	GM2A	PGD [d]	VCP[d]		C4BPA[d]
	CAPZB	GNB1	PGLYRP2	VPS35		CPN2
	CBR1	GNB2	PLG	VPS4B		FGA[d]
	CCT2	GNS	PLIN3	XRCC6		HRG[d]
	CCT5	GRN	PNP [d]	FLNB[a]		LRG1[d]
	CCT6A	GSN[d]	PPA1	CD44[a]		ORM1[d]
	CCT7	GSR	PPIB [d]	PRDX5[a]		SERPINA1[d]
	CD14	GSTP1[d]	PRDX2[d]	RAB7A[a]		SERPINA6[d]
	CFI	H1-5	PRDX6	RHOA[a]		SERPINA7[d]
	CLEC3B	HADHA	PRTN3[d]	RACK1[a]		VCL[d]
	CMPK1	HEBP1	PTPN6	FASN[b]		AZGP1[c,d]
				S100A6[b]		PRDX2 [c,d]
						TTR [c]

a Found in present study, breast cancer protein in UniProt database and Al-Wajeeh et al. (2020)

b Found in present study, Al-Wajeeh et al. (2020) and Neagu et al. (2022).

c Found in present study and Sinha et al. (2023).

d Found in present study, Al-Wajeeh et al. (2020) and Sinha et al. (2023).

*Found in both breast tissue (Al-Wajeeh et al., 2020) and urine (Beretov et al., 2015) samples.

Table S4. Differentially expressed proteins based on pairwise comparisons between breast cancer (BC) and Benign breast disease (BBD), symptom controls (SC) and healthy control (HC). Fold-change (FC) threshold 2 and false-discovery rate 0.05.

BC vs	Protein	FC	log2(FC)	p.adjusted	-log10 (p)	Expression
BBD	peroxiredoxin-6	0.034097	-4.8742	2.16E-25	24.666	Down
	rab GDP dissociation inhibitor beta isoform 1	0.045116	-4.4702	1.37E-22	21.863	Down
	heterogeneous nuclear ribonucleoprotein H isoform c	0.025347	-5.302	5.47E-20	19.262	Down
	ERO1-like protein alpha isoform 2 precursor	0.029305	-5.0927	7.69E-20	19.114	Down
	cadherin-1 isoform 1 preproprotein	35.737	5.1593	1.50E-19	18.825	Up
	ganglioside GM2 activator isoform 1 precursor	89.5	6.4838	1.02E-18	17.991	Up
	fibrinogen gamma chain isoform gamma-B precursor	235.93	7.8822	1.52E-15	14.818	Up
	polyunsaturated fatty acid lipoxygenase ALOX12	0.018946	-5.7219	2.74E-14	13.562	Down
	neprilysin isoform X1	0.041311	-4.5973	2.66E-13	12.575	Down
	ribonuclease inhibitor	0.018666	-5.7434	3.04E-13	12.517	Down
	transgelin-2 isoform a	0.0079612	-6.9728	3.96E-13	12.402	Down
	T-complex protein 1 subunit alpha isoform a	0.054712	-4.192	8.01E-13	12.097	Down
	complement component C9 preproprotein	53.204	5.7335	6.42E-12	11.192	Up
	intraflagellar transport protein 80 homolog isoform a	0.035993	-4.7962	7.67E-12	11.115	Down
	aurora kinase C isoform 1	27.365	4.7742	7.67E-12	11.115	Up
	aldo-keto reductase family 1 member C3 isoform 2	0.10537	-3.2465	9.20E-12	11.036	Down
	actin-related protein 2/3 complex subunit 1A isoform 1	0.073396	-3.7682	9.50E-12	11.022	Down
	beta-1,4-glucuronyltransferase 1	86.879	6.4409	1.03E-11	10.988	Up
	desmoglein-1 preproprotein	0.061202	-4.0303	1.03E-11	10.988	Down
	complement C1r subcomponent-like protein isoform 1 precursor	21.986	4.4585	1.07E-11	10.97	Up
	beta-glucuronidase isoform 1 precursor	37.256	5.2194	1.49E-11	10.828	Up
	alpha-actinin-1 isoform X9	66.38	6.0527	1.72E-11	10.766	Up
	thioredoxin domain-containing protein 17	0.089753	-3.4779	3.23E-11	10.491	Down
	catenin beta-1 isoform X1	0.024415	-5.3561	5.43E-11	10.265	Down
	phosphate carrier protein, mitochondrial isoform a precursor	55.741	5.8007	5.66E-11	10.247	Up

	heterogeneous nuclear ribonucleoprotein Q isoform X1	0.12171	-3.0385	1.01E-10	9.9942	Down
	cadherin-11 isoform 1 preproprotein	46.649	5.5438	1.06E-10	9.9764	Up
	tubulin beta-6 chain isoform 1	0.0097097	-6.6864	1.20E-10	9.9219	Down
	protein TESPA1 isoform X1	0.0050262	-7.6363	1.39E-10	9.8558	Down
	neutrophil elastase preproprotein	103.08	6.6877	2.01E-10	9.696	Up
	collagen alpha-1(XV) chain isoform X1	54.361	5.7645	2.15E-10	9.6672	Up
	glucose-6-phosphate 1-dehydrogenase isoform a	0.026555	-5.2349	3.36E-10	9.4734	Down
	mucin-1 isoform 22 precursor	33.327	5.0586	4.23E-10	9.3733	Up
	solute carrier family 12 member 3 isoform 1	0.077168	-3.6959	4.91E-10	9.309	Down
	annexin A4 isoform X1	0.04153	-4.5897	5.31E-10	9.2746	Down
	argininosuccinate synthase	15.305	3.936	5.31E-10	9.2746	Up
	peptidyl-glycine alpha-amidating monooxygenase isoform X1	0.11482	-3.1225	5.31E-10	9.2746	Down
	E3 ubiquitin-protein ligase TRIM4 isoform alpha	0.15466	-2.6929	5.31E-10	9.2746	Down
	apolipoprotein A-IV precursor	102.03	6.6728	5.94E-10	9.226	Up
	glutathione S-transferase Mu 2 isoform 1	0.088053	-3.5055	6.66E-10	9.1762	Down
	peptidase inhibitor 16 precursor	47.274	5.563	7.55E-10	9.1218	Up
	catenin delta-1 isoform 1ABC	0.083541	-3.5814	8.91E-10	9.05	Down
	cysteine-rich C-terminal protein 1 isoform X1	24.244	4.5996	9.98E-10	9.001	Up
	multiple epidermal growth factor-like domains protein 8 isoform 1 precursor	0.079268	-3.6571	1.17E-09	8.9315	Down
	guanine nucleotide-binding protein G(I)/G(S)/G(T) subunit beta-1 isoform X1	18.045	4.1736	1.35E-09	8.8687	Up
	vacuolar protein sorting-associated protein 4B	0.15634	-2.6773	1.98E-09	8.7025	Down
	40S ribosomal protein S16 isoform 1	0.11643	-3.1025	2.57E-09	8.5903	Down
	lactoylglutathione lyase	0.17095	-2.5484	2.71E-09	8.5663	Down
	transaldolase	13.73	3.7793	3.01E-09	8.5218	Up
	tripartite motif-containing protein 29 isoform X1	0.11791	-3.0842	3.12E-09	8.5053	Down
	lithostathine-1-alpha precursor	59.408	5.8926	3.55E-09	8.4496	Up
	myosin-14 isoform 3	0.032014	-4.9651	3.86E-09	8.4135	Down
	glia maturation factor gamma isoform 1	0.099147	-3.3343	3.86E-09	8.4135	Down
	vascular cell adhesion protein 1 isoform a precursor	19.152	4.2594	5.21E-09	8.2833	Up
	ATP-dependent translocase ABCB1 isoform 1	19.589	4.292	8.92E-09	8.0495	Up

	tumor protein p63-regulated gene 1 protein isoform X1	19.589	4.292	8.92E-09	8.0495	Up
	40S ribosomal protein S2	0.061994	-4.0117	9.71E-09	8.0127	Down
	hepcidin preproprotein	0.0079809	-6.9692	9.82E-09	8.0077	Down
	BRO1 domain-containing protein BROX isoform X1	0.13108	-2.9315	1.08E-08	7.9648	Down
	protein NDRG1 isoform 4	0.16521	-2.5977	1.13E-08	7.9455	Down
	heme-binding protein 1	0.16872	-2.5673	1.45E-08	7.8396	Down
	syntenin-1 isoform 7	0.026791	-5.2221	1.62E-08	7.7905	Down
	alpha-1B-glycoprotein precursor	6.6686	2.7374	1.62E-08	7.7905	Up
	tyrosine-protein phosphatase non-receptor type 6 isoform 3	0.0142	-6.138	1.68E-08	7.775	Down
	serine/threonine-protein kinase 24 isoform a precursor	0.094174	-3.4085	1.68E-08	7.775	Down
	vacuolar protein sorting-associated protein 35	0.13142	-2.9278	2.15E-08	7.667	Down
	hemicentin-1 precursor	19.791	4.3068	2.29E-08	7.6407	Up
	endosialin precursor	23.652	4.5639	2.92E-08	7.5348	Up
	methyltransferase-like protein 7A precursor	0.1592	-2.6511	3.16E-08	7.5009	Down
	cytosolic non-specific dipeptidase isoform X1	0.024263	-5.3651	3.41E-08	7.467	Down
	folate receptor alpha precursor	0.10288	-3.2809	3.41E-08	7.467	Down
	histidine-rich glycoprotein isoform X1	35.183	5.1368	3.69E-08	7.4334	Up
	suprabasin isoform X1	0.037435	-4.7395	3.80E-08	7.4203	Down
	T-complex protein 1 subunit epsilon isoform a	0.039701	-4.6547	3.80E-08	7.4203	Down
	ATP synthase subunit gamma, mitochondrial isoform X1	19.791	4.3067	4.08E-08	7.3897	Up
	toll-interacting protein isoform 1	0.092131	-3.4402	4.10E-08	7.3869	Down
	X-ray repair cross-complementing protein 6 isoform 1	0.18514	-2.4333	4.10E-08	7.3869	Down
	purine nucleoside phosphorylase	0.17065	-2.5509	4.36E-08	7.3608	Down
	ubiquitin thioesterase OTU1 isoform 1	0.091819	-3.4451	4.44E-08	7.3526	Down
	annexin A8-like protein 1 isoform 1	0.16491	-2.6002	4.48E-08	7.3486	Down
	fibrinogen beta chain isoform 1 preproprotein	62.29	5.9609	4.66E-08	7.3313	Up
	serine/threonine-protein phosphatase 2A 55 kDa regulatory subunit B beta isoform isoform a	0.17581	-2.5079	4.75E-08	7.3229	Down
	60S ribosomal protein L11 isoform 1	0.094339	-3.406	5.03E-08	7.2981	Down
	protein S100-A2 isoform 1	15.783	3.9803	5.77E-08	7.239	Up
	myosin regulatory light chain 12A isoform 2	0.12268	-3.0271	5.98E-08	7.2235	Down

	40S ribosomal protein SA isoform 2	0.041732	-4.5827	6.05E-08	7.2184	Down
	cytochrome b-245 heavy chain	0.069038	-3.8565	8.02E-08	7.0958	Down
	hairy and enhancer of split-related protein HELT isoform X1	142.22	7.152	8.42E-08	7.0745	Up
	neutral alpha-glucosidase AB isoform 3 precursor	57.271	5.8397	8.74E-08	7.0584	Up
	tuftelin-interacting protein 11 isoform 1	0.022293	-5.4873	9.83E-08	7.0074	Down
	macrophage colony-stimulating factor 1 isoform a precursor	0.092078	-3.441	1.13E-07	6.9463	Down
	40S ribosomal protein S26	0.040331	-4.632	1.20E-07	6.9213	Down
	destrin isoform a	17.175	4.1022	1.30E-07	6.887	Up
	hepatitis A virus cellular receptor 2 precursor	0.047361	-4.4001	1.47E-07	6.8325	Down
	major prion protein preproprotein Prp precursor	11.116	3.4745	1.52E-07	6.8186	Up
	serine/threonine-protein kinase MRCK alpha isoform F	10.063	3.331	1.56E-07	6.808	Up
	N(G),N(G)-dimethylarginine dimethylaminohydrolase 2 isoform X1	20.713	4.3725	1.62E-07	6.7905	Up
	corticosteroid-binding globulin isoform X1	35.75	5.1599	1.75E-07	6.7569	Up
	maltase-glucoamylase isoform X1	16.255	4.0228	1.76E-07	6.7554	Up
	nectin-2 isoform delta precursor	15.468	3.9512	1.93E-07	6.7135	Up
	general vesicular transport factor p115 isoform X1	30.466	4.9291	1.96E-07	6.708	Up
	ras-related protein Rab-7a	12.569	3.6517	2.06E-07	6.687	Up
	N-acetylmuramoyl-L-alanine amidase isoform 2 precursor	4.4797	2.1634	2.62E-07	6.5818	Up
	ADP-ribosylation factor-like protein 1 isoform 1	0.050474	-4.3083	2.64E-07	6.5786	Down
	enoyl-CoA hydratase, mitochondrial	7.3817	2.884	2.86E-07	6.5439	Up
	aldehyde dehydrogenase, mitochondrial isoform 1 precursor	9.5288	3.2523	4.75E-07	6.323	Up
	immunoglobulin J chain precursor	5.7908	2.5338	5.46E-07	6.2628	Up
	N-acetylglucosamine-6-sulfatase precursor	15.591	3.9626	5.84E-07	6.2338	Up
	collagen alpha-2(I) chain precursor	29.94	4.904	5.97E-07	6.2237	Up
	15-hydroxyprostaglandin dehydrogenase [NAD(+)] isoform 1	14.789	3.8864	6.21E-07	6.2067	Up
	N-sulphoglucosamine sulphohydrolase isoform 1 precursor	11.749	3.5545	6.70E-07	6.1738	Up

	delta-1-pyrroline-5-carboxylate dehydrogenase, mitochondrial isoform a precursor	19.026	4.2499	7.40E-07	6.131	Up
	prostatic acid phosphatase isoform PAP precursor	6.8413	2.7743	8.73E-07	6.0588	Up
	plasminogen activator inhibitor 2	8.8257	3.1417	9.71E-07	6.0127	Up
	acid ceramidase isoform b	11.405	3.5115	9.91E-07	6.0041	Up
	receptor of activated protein C kinase 1	8.746	3.1286	9.97E-07	6.0012	Up
	apolipoprotein E isoform a precursor	19.245	4.2664	1.10E-06	5.9578	Up
	latent-transforming growth factor beta-binding protein 2 precursor	23.298	4.5421	1.31E-06	5.8836	Up
	CDK5 regulatory subunit-associated protein 3 isoform c	17.34	4.116	1.31E-06	5.8836	Up
	sialate O-acetylesterase isoform 1 precursor	13.747	3.7811	1.36E-06	5.8651	Up
	dystroglycan 1 isoform X1	17.372	4.1187	1.41E-06	5.852	Up
	60S ribosomal protein L4	20.115	4.3302	1.78E-06	5.7494	Up
	phosphomevalonate kinase isoform 1	27.487	4.7807	2.03E-06	5.6934	Up
	glutathione reductase, mitochondrial isoform 1 precursor	43.834	5.454	2.10E-06	5.6776	Up
	beta/gamma crystallin domain-containing protein 1 isoform X1	22.254	4.476	2.14E-06	5.6689	Up
	chondroitin sulfate proteoglycan 4 precursor	9.4323	3.2376	2.32E-06	5.6343	Up
	apolipoprotein A-II preproprotein	63.559	5.99	2.39E-06	5.6213	Up
	pregnancy-specific beta-1-glycoprotein 11 isoform 1 precursor	18.396	4.2013	2.39E-06	5.6213	Up
	bone marrow proteoglycan isoform 1 preproprotein	37.475	5.2279	2.80E-06	5.5521	Up
	dipeptidyl peptidase 2 preproprotein	7.4836	2.9037	2.95E-06	5.5307	Up
	rho GDP-dissociation inhibitor 2	54.357	5.7644	3.54E-06	5.4512	Up
	annexin A6 isoform 1	11.971	3.5815	3.73E-06	5.4282	Up
	mucin-5B precursor	11.971	3.5815	3.73E-06	5.4282	Up
	gelsolin isoform b	2.4546	1.2955	4.23E-06	5.3737	Up
	tripeptidyl-peptidase 1 preproprotein	6.1687	2.625	5.70E-06	5.2442	Up
	TBC1 domain family member 9	2.836	1.5039	5.70E-06	5.244	Up
	gamma-glutamyl hydrolase precursor	5.4854	2.4556	9.86E-06	5.006	Up
	aminopeptidase N	2.7338	1.4509	1.45E-05	4.8387	Up
	alpha-1-acid glycoprotein 1 precursor	6.8083	2.7673	4.49E-05	4.3476	Up
	lamin isoform A	0.25345	-1.9802	5.10E-05	4.2925	Down

	carbonyl reductase [NADPH] 1 isoform 1	0.20239	-2.3048	6.33E-05	4.1984	Down
	zinc-alpha-2-glycoprotein precursor	3.4958	1.8056	0.00011996	3.921	Up
	prostaglandin-H2 D-isomerase precursor	2.479	1.3098	0.00014276	3.8454	Up
	40S ribosomal protein S3 isoform 2	0.33298	-1.5865	0.00027437	3.5617	Down
	catechol O-methyltransferase isoform MB-COMT	0.31113	-1.6844	0.0003168	3.4992	Down
	endonuclease domain-containing 1 protein precursor	3.0021	1.586	0.00037726	3.4234	Up
	superoxide dismutase [Mn], mitochondrial isoform A precursor	3.5596	1.8317	0.00041841	3.3784	Up
	protein AMBP preproprotein	2.5254	1.3365	0.00049505	3.3053	Up
	aldo-keto reductase family 1 member A1	0.40508	-1.3037	0.00060713	3.2167	Down
	ras GTPase-activating-like protein IQGAP1	0.4136	-1.2737	0.00079161	3.1015	Down
	immunoglobulin lambda-like polypeptide 5 isoform 1	2.3642	1.2413	0.00089375	3.0488	Up
	histone H3.2	0.36632	-1.4488	0.0011702	2.9318	Down
	proline-rich protein 11 isoform X1	3.8257	1.9357	0.0013208	2.8792	Up
	multimerin-2 precursor	2.5428	1.3464	0.001712	2.7665	Up
	guanylate-binding protein 6 isoform 1	0.4004	-1.3205	0.001712	2.7665	Down
	alpha-N-acetylglucosaminidase isoform X1	3.0801	1.623	0.001873	2.7275	Up
	thyroxine-binding globulin isoform X1	3.7362	1.9016	0.0019183	2.7171	Up
	60S acidic ribosomal protein P0	0.40799	-1.2934	0.0019418	2.7118	Down
	complement C4-A isoform 1 preproprotein	2.0508	1.0362	0.0021788	2.6618	Up
	alpha-1-acid glycoprotein 2 precursor	3.2397	1.6959	0.0023375	2.6312	Up
	aquaporin-1 isoform 5	4.062	2.0222	0.0024853	2.6046	Up
	olfactomedin-4 precursor	2.0169	1.0121	0.0045587	2.3412	Up
	F-actin-capping protein subunit beta isoform X1	0.38847	-1.3641	0.0048461	2.3146	Down
	ubiquitin-like modifier-activating enzyme 1 isoform X1	2.022	1.0158	0.0049524	2.3052	Up
	adenine phosphoribosyltransferase isoform a	0.21623	-2.2094	0.0051869	2.2851	Down
	D-3-phosphoglycerate dehydrogenase isoform X1	0.35617	-1.4894	0.005604	2.2515	Down
	vesicular integral-membrane protein VIP36 precursor	2.257	1.1744	0.0061018	2.2145	Up
	ADP/ATP translocase 2	11.138	3.4774	0.0078045	2.1077	Up
	plastin-3 isoform X1	0.3157	-1.6634	0.0079167	2.1015	Down

	mannosyl-oligosaccharide 1,2-alpha-mannosidase IA isoform X1	3.2534	1.702	0.0084707	2.0721	Up
	protein MMS22-like isoform X1	0.35078	-1.5114	0.010441	1.9813	Down
	flavin reductase (NADPH)	0.44031	-1.1834	0.012583	1.9002	Down
	chloride intracellular channel protein 3	0.48991	-1.0294	0.015475	1.8104	Down
	protein-glutamine gamma-glutamyltransferase K	0.42172	-1.2456	0.01582	1.8008	Down
	low-density lipoprotein receptor-related protein 2 precursor	2.4733	1.3064	0.017546	1.7558	Up
	transthyretin precursor	2.3979	1.2618	0.018464	1.7337	Up
	ceruloplasmin isoform X1	2.3877	1.2556	0.018725	1.7276	Up
	plasminogen isoform 1 precursor	2.0432	1.0308	0.019322	1.714	Up
	ras-related protein Rab-5A isoform 1	0.47357	-1.0784	0.023461	1.6296	Down
	major vault protein isoform 1	3.7987	1.9255	0.025856	1.5874	Up
	EGF-containing fibulin-like extracellular matrix protein 1 precursor	2.1589	1.1103	0.027366	1.5628	Up
	perilipin-3 isoform 1	0.40181	-1.3154	0.031481	1.5019	Down
	voltage-dependent anion-selective channel protein 1 isoform 6	2.7152	1.4411	0.033252	1.4782	Up
	ABHD14A-ACY1 readthrough (NMD candidate)	0.32188	-1.6354	0.034442	1.4629	Down
	cathepsin B isoform 1 preproprotein	2.0789	1.0558	0.037473	1.4263	Up
	hemopexin precursor	2.9789	1.5748	0.040217	1.3956	Up
	myosin-9	2.2042	1.1402	0.045252	1.3444	Up
	vitamin D-binding protein isoform 3 precursor	2.0092	1.0066	0.047939	1.3193	Up
SC	N-acetylmuramoyl-L-alanine amidase isoform 2 precursor	99.308	6.6338	2.74E-20	19.563	Up
	ERO1-like protein alpha isoform 2 precursor	0.026479	-5.239	2.74E-20	19.563	Down
	ganglioside GM2 activator isoform 1 precursor	131.14	7.035	6.38E-17	16.195	Up
	ribonuclease inhibitor	0.005662	-7.4645	2.61E-16	15.584	Down
	dipeptidyl peptidase 2 preproprotein	63.118	5.98	2.89E-15	14.539	Up
	sulfhydryl oxidase 1 isoform a precursor	0.032503	-4.9433	3.42E-15	14.466	Down
	polyunsaturated fatty acid lipoxygenase ALOX12	0.067794	-3.8827	3.42E-15	14.466	Down
	heterogeneous nuclear ribonucleoprotein Q isoform X1	0.066129	-3.9186	6.14E-14	13.212	Down
	fibrinogen gamma chain isoform gamma-B precursor	235.93	7.8822	1.61E-13	12.793	Up
	peroxiredoxin-6	0.032014	-4.9651	1.61E-13	12.793	Down

	rab GDP dissociation inhibitor beta isoform 1	0.033017	-4.9207	1.78E-13	12.75	Down
	desmoglein-1 preproprotein	0.096135	-3.3788	3.93E-13	12.406	Down
	T-complex protein 1 subunit alpha isoform a	0.062649	-3.9966	6.07E-13	12.217	Down
	T-complex protein 1 subunit beta isoform 1	0.057486	-4.1207	1.13E-11	10.948	Down
	solute carrier family 12 member 3 isoform 1	0.083444	-3.5831	1.16E-11	10.937	Down
	protein TESPA1 isoform X1	0.041639	-4.5859	2.95E-11	10.529	Down
	annexin A8-like protein 1 isoform 1	0.075585	-3.7258	2.95E-11	10.529	Down
	translationally-controlled tumor protein isoform 1	0.029833	-5.0669	7.17E-11	10.144	Down
	catenin delta-1 isoform 1ABC	0.12769	-2.9692	7.27E-11	10.139	Down
	neprilysin isoform X1	0.045594	-4.455	9.90E-11	10.004	Down
	collagen alpha-3(VI) chain isoform 1 precursor	0.043507	-4.5226	3.78E-10	9.4223	Down
	major vault protein isoform 1	69.755	6.1242	3.82E-10	9.418	Up
	heterogeneous nuclear ribonucleoprotein H isoform c	0.03273	-4.9332	5.31E-10	9.2748	Down
	beta-glucuronidase isoform 1 precursor	37.256	5.2194	5.81E-10	9.236	Up
	alpha-actinin-1 isoform X9	66.38	6.0527	6.33E-10	9.1986	Up
	succinate--CoA ligase [ADP/GDP-forming] subunit alpha, mitochondrial precursor	0.11275	-3.1488	6.33E-10	9.1986	Down
	60S ribosomal protein L11 isoform 1	0.018732	-5.7383	6.57E-10	9.1827	Down
	membrane protein FAM174A isoform X1	0.022566	-5.4697	6.74E-10	9.1712	Down
	protein NDRG1 isoform 4	0.075954	-3.7187	6.74E-10	9.1712	Down
	cadherin-11 isoform 1 preproprotein	61.141	5.9341	8.35E-10	9.0786	Up
	ammonium transporter Rh type C	0.075274	-3.7317	9.46E-10	9.024	Down
	trefoil factor 2 precursor	104.77	6.711	1.20E-09	8.9203	Up
	collagen alpha-1(XV) chain isoform X1	85.432	6.4167	1.29E-09	8.8898	Up
	phosphate carrier protein, mitochondrial isoform a precursor	11.305	3.4989	1.49E-09	8.8264	Up
	elongation factor 1-delta isoform X1	0.11326	-3.1423	1.84E-09	8.7345	Down
	keratinocyte proline-rich protein	51.79	5.6946	1.86E-09	8.7299	Up
	actin-related protein 2/3 complex subunit 1A isoform 1	0.092051	-3.4414	1.96E-09	8.707	Down
	tubulin beta-6 chain isoform 1	0.11182	-3.1607	3.63E-09	8.4399	Down
	transgelin-2 isoform a	0.042547	-4.5548	4.44E-09	8.3526	Down
	40S ribosomal protein S24 isoform d	0.094758	-3.3996	4.44E-09	8.3526	Down

	glucose-6-phosphate 1-dehydrogenase isoform a	0.071367	-3.8086	5.04E-09	8.2977	Down
	tetranectin isoform 1precursor	0.084686	-3.5617	5.95E-09	8.2252	Down
	protein NDRG2 isoform g	0.043404	-4.526	8.19E-09	8.0865	Down
	peptidyl-glycine alpha-amidating monooxygenase isoform X1	0.12165	-3.0392	1.56E-08	7.8069	Down
	3-mercaptopyruvate sulfurtransferase isoform 1	0.17095	-2.5484	1.56E-08	7.8069	Down
	peptidase inhibitor 16 precursor	47.274	5.563	1.60E-08	7.7947	Up
	cytosolic non-specific dipeptidase isoform X1	0.04836	-4.3701	1.60E-08	7.7947	Down
	hydroxysteroid dehydrogenase-like protein 2 isoform 1	0.049097	-4.3482	1.60E-08	7.7947	Down
	toll-interacting protein isoform 1	0.077362	-3.6922	1.60E-08	7.7947	Down
	F-actin-capping protein subunit alpha-1	0.11853	-3.0767	1.82E-08	7.7397	Down
	lactoylglutathione lyase	0.10546	-3.2453	1.96E-08	7.7082	Down
	cysteine-rich C-terminal protein 1 isoform X1	24.244	4.5996	1.97E-08	7.7047	Up
	vacuolar protein sorting-associated protein 35	0.095703	-3.3853	2.45E-08	7.6105	Down
	guanine nucleotide-binding protein G(I)/G(S)/G(T) subunit beta-1 isoform X1	18.045	4.1736	2.58E-08	7.5887	Up
	40S ribosomal protein S12	0.064075	-3.9641	2.83E-08	7.5478	Down
	receptor-type tyrosine-protein phosphatase S isoform 1 precursor	0.036633	-4.7707	2.85E-08	7.5457	Down
	myosin regulatory light chain 12A isoform 2	0.052259	-4.2582	2.85E-08	7.5457	Down
	astrocytic phosphoprotein PEA-15 isoform b	0.025861	-5.2731	4.17E-08	7.3801	Down
	multiple epidermal growth factor-like domains protein 8 isoform 1 precursor	0.018143	-5.7844	4.45E-08	7.3518	Down
	epidermal growth factor receptor kinase substrate 8-like protein 2 isoform X1	0.098218	-3.3479	5.45E-08	7.2637	Down
	lithostathine-1-alpha precursor	59.408	5.8926	5.87E-08	7.2314	Up
	T-complex protein 1 subunit zeta isoform a	0.085772	-3.5434	5.87E-08	7.2314	Down
	carcinoembryonic antigen-related cell adhesion molecule 5 isoform 1 preproprotein	0.0073142	-7.0951	6.93E-08	7.1591	Down
	spectrin alpha chain, erythrocytic 1 isoform X1	0.037742	-4.7277	7.58E-08	7.1202	Down
	vascular cell adhesion protein 1 isoform a precursor	19.152	4.2594	8.10E-08	7.0915	Up

	putative V-set and immunoglobulin domain-containing-like protein IGHV4OR15-8	0.064146	-3.9625	9.53E-08	7.021	Down
	protein-glutamine gamma-glutamyltransferase E	0.07988	-3.646	9.89E-08	7.005	Down
	epiplakin isoform X4	0.050949	-4.2948	9.90E-08	7.0044	Down
	nucleoside diphosphate kinase A isoform a	0.006312	-7.3077	1.15E-07	6.94	Down
	myosin-14 isoform 3	0.047558	-4.3942	1.19E-07	6.9255	Down
	ATP-dependent translocase ABCB1 isoform 1	19.589	4.292	1.20E-07	6.9194	Up
	tumor protein p63-regulated gene 1 protein isoform X1	19.589	4.292	1.20E-07	6.9194	Up
	neutrophil cytosol factor 2 isoform X1	0.016294	-5.9395	1.40E-07	6.853	Down
	biglycan preproprotein	0.0088953	-6.8127	2.44E-07	6.6122	Down
	hemicentin-1 precursor	19.791	4.3068	3.05E-07	6.5153	Up
	extracellular superoxide dismutase [Cu-Zn] preproprotein	0.019932	-5.6488	3.15E-07	6.502	Down
	beta-2-glycoprotein 1 precursor	11.448	3.517	4.43E-07	6.3537	Up
	histidine-rich glycoprotein isoform X1	35.183	5.1368	4.72E-07	6.3256	Up
	ABHD14A-ACY1 readthrough (NMD candidate)	11.428	3.5145	5.02E-07	6.299	Up
	complement factor I isoform 4 preproprotein	0.0087628	-6.8344	5.74E-07	6.2411	Down
	folate receptor alpha precursor	0.02032	-5.621	5.89E-07	6.2297	Down
	fibrinogen beta chain isoform 1 preproprotein	62.29	5.9609	6.10E-07	6.2148	Up
	rabphilin-3A isoform 1	12.861	3.6849	9.46E-07	6.0241	Up
	hairy and enhancer of split-related protein HELT isoform X1	142.22	7.152	1.07E-06	5.9721	Up
	neutral alpha-glucosidase AB isoform 3 precursor	57.271	5.8397	1.10E-06	5.959	Up
	aldo-keto reductase family 1 member A1	8.5859	3.102	1.53E-06	5.8159	Up
	plastin-3 isoform X1	13.536	3.7587	1.58E-06	5.8023	Up
	major prion protein preproprotein Prp precursor	11.116	3.4745	1.81E-06	5.7425	Up
	adipocyte plasma membrane-associated protein	0.0029862	-8.3875	1.83E-06	5.7366	Down
	corticosteroid-binding globulin isoform X1	35.75	5.1599	2.05E-06	5.6874	Up
	maltase-glucoamylase isoform X1	16.255	4.0228	2.06E-06	5.687	Up
	TBC1 domain family member 8 isoform 1	23.889	4.5783	2.16E-06	5.6664	Up
	nectin-2 isoform delta precursor	15.468	3.9512	2.21E-06	5.6566	Up
	ras-related protein Rab-7a	12.569	3.6517	2.34E-06	5.6309	Up

	enoyl-CoA hydratase, mitochondrial	7.3817	2.884	3.15E-06	5.5012	Up
	malate dehydrogenase, cytoplasmic isoform 2	7.0746	2.8226	3.99E-06	5.3992	Up
	chondroitin sulfate proteoglycan 4 precursor	13.258	3.7288	4.13E-06	5.3839	Up
	vinculin isoform meta-VCL	12.588	3.654	4.13E-06	5.3839	Up
	aldehyde dehydrogenase, mitochondrial isoform 1 precursor	9.5288	3.2523	4.73E-06	5.325	Up
	pregnancy-specific beta-1-glycoprotein 11 isoform 1 precursor	28.591	4.8375	4.75E-06	5.323	Up
	acid ceramidase isoform b	10.115	3.3384	4.75E-06	5.323	Up
	arf-GAP with Rho-GAP domain, ANK repeat and PH domain-containing protein 1 isoform c	65.864	6.0414	5.28E-06	5.2776	Up
	beta-galactosidase isoform d precursor	39.894	5.3181	5.28E-06	5.277	Up
	N-acetylglucosamine-6-sulfatase precursor	15.591	3.9626	5.46E-06	5.2625	Up
	collagen alpha-2(I) chain precursor	29.94	4.904	5.56E-06	5.2545	Up
	annexin A6 isoform 1	18.38	4.2001	5.62E-06	5.25	Up
	mucin-5B precursor	18.38	4.2001	5.62E-06	5.25	Up
	15-hydroxyprostaglandin dehydrogenase [NAD(+)] isoform 1	14.789	3.8864	5.64E-06	5.2487	Up
	calpain small subunit 1 isoform X1	10.682	3.4172	5.79E-06	5.2373	Up
	N-sulphoglucosamine sulphohydrolase isoform 1 precursor	11.749	3.5545	5.96E-06	5.225	Up
	delta-1-pyrroline-5-carboxylate dehydrogenase, mitochondrial isoform a precursor	19.026	4.2499	6.48E-06	5.1887	Up
	heterogeneous nuclear ribonucleoprotein A1 isoform b	14.864	3.8938	6.68E-06	5.1755	Up
	40S ribosomal protein S3 isoform 2	10.981	3.457	6.75E-06	5.1709	Up
	adenine phosphoribosyltransferase isoform a	8.4773	3.0836	6.75E-06	5.1709	Up
	calpain-1 catalytic subunit isoform X1	0.37985	-1.3965	7.54E-06	5.1227	Down
	plasminogen activator inhibitor 2	8.8257	3.1417	7.96E-06	5.0993	Up
	receptor of activated protein C kinase 1	8.746	3.1286	8.19E-06	5.0866	Up
	renin receptor precursor	15.101	3.9166	8.62E-06	5.0643	Up
	latent-transforming growth factor beta-binding protein 2 precursor	23.298	4.5421	1.03E-05	4.9869	Up

	CDK5 regulatory subunit-associated protein 3 isoform c	17.34	4.116	1.03E-05	4.9869	Up
	sialate O-acetylesterase isoform 1 precursor	13.747	3.7811	1.06E-05	4.975	Up
	inorganic pyrophosphatase	12.919	3.6914	1.06E-05	4.975	Up
	dystroglycan 1 isoform X1	17.372	4.1187	1.09E-05	4.9642	Up
	60S ribosomal protein L4	20.115	4.3302	1.33E-05	4.8763	Up
	complement factor D isoform 2 precursor	20.501	4.3576	1.40E-05	4.8544	Up
	phosphomevalonate kinase isoform 1	27.487	4.7807	1.46E-05	4.8352	Up
	calmodulin-like protein 3	0.17669	-2.5007	1.46E-05	4.8352	Down
	glutathione reductase, mitochondrial isoform 1 precursor	43.834	5.454	1.51E-05	4.8222	Up
	beta/gamma crystallin domain-containing protein 1 isoform X1	22.254	4.476	1.53E-05	4.8152	Up
	apolipoprotein A-II preproprotein	63.559	5.99	1.70E-05	4.7706	Up
	cadherin-1 isoform 1 preproprotein	5.2629	2.3958	1.70E-05	4.77	Up
	superoxide dismutase [Mn], mitochondrial isoform A precursor	5.5294	2.4671	1.78E-05	4.75	Up
	L-xylulose reductase isoform 1	185.57	7.5358	2.03E-05	4.6916	Up
	rho GDP-dissociation inhibitor 2	54.357	5.7644	2.36E-05	4.627	Up
	complement C3 preproprotein	2.257	1.1744	7.10E-05	4.1487	Up
	zinc-alpha-2-glycoprotein precursor	3.5464	1.8263	8.67E-05	4.0619	Up
	alpha-1-acid glycoprotein 1 precursor	4.1534	2.0543	0.0001747	3.7577	Up
	D-3-phosphoglycerate dehydrogenase isoform X1	0.18111	-2.4651	0.00020321	3.6921	Down
	60S acidic ribosomal protein P0	0.30033	-1.7354	0.00024046	3.619	Down
	serpin B13 isoform 1	2.8397	1.5057	0.00033914	3.4696	Up
	catechol O-methyltransferase isoform MB-COMT	0.29261	-1.773	0.00036219	3.4411	Down
	alpha-1B-glycoprotein precursor	3.9368	1.977	0.00045633	3.3407	Up
	guanine nucleotide-binding protein G(I)/G(S)/G(T) subunit beta-2	0.36575	-1.4511	0.00059788	3.2234	Down
	endoplasmic reticulum chaperone BiP precursor	0.194	-2.3659	0.00074058	3.1304	Down
	proline-rich protein 11 isoform X1	4.5035	2.171	0.00079505	3.0996	Up
	CD44 antigen isoform X1	2.5512	1.3512	0.00079609	3.099	Up
	prostaglandin-H2 D-isomerase precursor	2.5396	1.3446	0.00081696	3.0878	Up
	immunoglobulin J chain precursor	3.2984	1.7218	0.001162	2.9348	Up

	heat shock protein HSP 90-alpha isoform 1	2.9154	1.5437	0.0014375	2.8424	Up
	multimerin-2 precursor	2.9436	1.5576	0.0015414	2.8121	Up
	SH3 domain-binding glutamic acid-rich-like protein 3	2.3135	1.2101	0.001699	2.7698	Up
	cathepsin B isoform 1 preproprotein	3.1395	1.6505	0.0018413	2.7349	Up
	ubiquitin-like modifier-activating enzyme 1 isoform X1	2.4999	1.3219	0.0020945	2.6789	Up
	programmed cell death 6-interacting protein isoform 2	0.43244	-1.2094	0.0029976	2.5232	Down
	perilipin-3 isoform 1	0.43109	-1.2139	0.0032436	2.489	Down
	carbonyl reductase [NADPH] 1 isoform 1	0.20532	-2.2841	0.0047803	2.3205	Down
	zinc finger protein 185 isoform X7	0.20787	-2.2663	0.004927	2.3074	Down
	endonuclease domain-containing 1 protein precursor	4.3829	2.1319	0.0052498	2.2799	Up
	ceruloplasmin isoform X1	3.0205	1.5948	0.0077934	2.1083	Up
	immunoglobulin lambda-like polypeptide 5 isoform 1	2.3922	1.2583	0.0079704	2.0985	Up
	plakophilin-1 isoform 1b	0.48867	-1.0331	0.008503	2.0704	Down
	EGF-containing fibulin-like extracellular matrix protein 1 precursor	2.042	1.03	0.011669	1.933	Up
	ras-related protein Rab-1B	0.33444	-1.5802	0.017573	1.7552	Down
	progranulin precursor	4.2326	2.0815	0.018391	1.7354	Up
	neutrophil elastase preproprotein	4.8532	2.2789	0.02822	1.5494	Up
	peroxiredoxin-5, mitochondrial isoform L precursor	3.4419	1.7832	0.028874	1.5395	Up
	neutrophil gelatinase-associated lipocalin isoform X1	3.0724	1.6194	0.030275	1.5189	Up
	alpha-N-acetylglucosaminidase isoform X1	2.1638	1.1136	0.031811	1.4974	Up
	UMP-CMP kinase isoform c	0.4854	-1.0427	0.033735	1.4719	Down
	guanylate-binding protein 6 isoform 1	0.4655	-1.1031	0.03666	1.4358	Down
	TBC1 domain family member 9	2.2023	1.139	0.040247	1.3953	Up
	neutrophil defensin 1 isoform 1 preproprotein	2.2667	1.1806	0.043117	1.3654	Up
	protein-glutamine gamma-glutamyltransferase K	0.11785	-3.085	0.046241	1.335	Down
HC	plasminogen isoform 1 precursor	122.78	6.94	2.1011E-14	13.678	Up
	alpha-1-acid glycoprotein 2 precursor	131.68	7.0408	1.5004E-11	10.824	Up
	ribonuclease inhibitor	0.040337	-4.6317	1.5004E-11	10.824	Down
	alpha-N-acetylglucosaminidase isoform X1	96.559	6.5933	1.5861E-11	10.8	Up

	protein-glutamine gamma-glutamyltransferase E	0.021256	-5.556	2.6798E-11	10.572	Down
	dermatopontin precursor	0.052979	-4.2384	2.0808E-10	9.6818	Down
	agrin isoform X1	39.358	5.2986	5.1194E-10	9.2908	Up
	syntenin-1 isoform 7	0.0081562	-6.9379	9.1365E-10	9.0392	Down
	leucine-rich alpha-2-glycoprotein precursor	273.31	8.0944	1.215E-09	8.9154	Up
	aldo-keto reductase family 1 member B10	61.08	5.9326	1.215E-09	8.9154	Up
	stAR-related lipid transfer protein 13 isoform X1	28.789	4.8474	2.5824E-09	8.588	Up
	histone H1.5	50.73	5.6648	5.6587E-09	8.2473	Up
	fibrinogen gamma chain isoform gamma-B precursor	235.93	7.8822	5.699E-09	8.2442	Up
	heterogeneous nuclear ribonucleoprotein Q isoform X1	0.077745	-3.6851	8.1974E-09	8.0863	Down
	protein-arginine deiminase type-2	0.0091832	-6.7668	1.5026E-08	7.8232	Down
	acetyl-CoA acetyltransferase, mitochondrial isoform a precursor	0.014569	-6.101	1.7068E-08	7.7678	Down
	ras-related protein Rab-3D	0.049355	-4.3407	1.9492E-08	7.7101	Down
	filamin-B isoform 1	0.1138	-3.1354	3.3795E-08	7.4711	Down
	aquaporin-1 isoform 5	77.418	6.2746	3.6482E-08	7.4379	Up
	sodium/potassium-transporting ATPase subunit alpha-1 isoform a	0.11357	-3.1383	4.8324E-08	7.3158	Down
	alpha-2-antiplasmin isoform X2	0.09455	-3.4028	5.8486E-08	7.233	Down
	BRO1 domain-containing protein BROX isoform X1	0.12708	-2.9761	5.8486E-08	7.233	Down
	vitamin D-binding protein isoform 3 precursor	29.857	4.9	5.9625E-08	7.2246	Up
	V-type proton ATPase catalytic subunit A isoform X1	0.14	-2.8365	5.9625E-08	7.2246	Down
	solute carrier family 12 member 3 isoform 1	0.077886	-3.6825	9.6636E-08	7.0149	Down
	myosin-14 isoform 3	0.042905	-4.5427	1.411E-07	6.8505	Down
	rab GDP dissociation inhibitor beta isoform 1	0.0046401	-7.7516	1.5761E-07	6.8024	Down
	endonuclease domain-containing 1 protein precursor	26.901	4.7496	1.5761E-07	6.8024	Up
	E3 ubiquitin/ISG15 ligase TRIM25	0.030798	-5.021	1.7433E-07	6.7586	Down
	neprilysin isoform X1	0.057728	-4.1146	1.7433E-07	6.7586	Down
	T-complex protein 1 subunit beta isoform 1	0.039695	-4.6549	2.1873E-07	6.6601	Down
	cytosolic purine 5'-nucleotidase isoform 2	0.013255	-6.2373	2.2157E-07	6.6545	Down
	C4b-binding protein alpha chain isoform X1	0.031675	-4.9805	2.4813E-07	6.6053	Down

	protein-glutamine gamma-glutamyltransferase 5 isoform 1	0.041711	-4.5834	2.4813E-07	6.6053	Down
	peroxiredoxin-6	0.15809	-2.6612	2.4813E-07	6.6053	Down
	tubulin beta-6 chain isoform 1	0.047251	-4.4035	2.774E-07	6.5569	Down
	calmodulin-3 isoform 1	0.11315	-3.1436	3.7011E-07	6.4317	Down
	serine/threonine-protein phosphatase 2A 65 kDa regulatory subunit A alpha isoform isoform 1	687.42	9.425	4.0757E-07	6.3898	Up
	heterogeneous nuclear ribonucleoprotein H isoform c	0.12357	-3.0166	4.1414E-07	6.3829	Down
	protein S100-A7	0.058489	-4.0957	4.5458E-07	6.3424	Down
	neutrophil gelatinase-associated lipocalin isoform X1	29.38	4.8768	4.8919E-07	6.3105	Up
	elongation factor 1-delta isoform X1	0.14189	-2.8172	4.9358E-07	6.3066	Down
	fibronectin isoform 1 precursor	3.0436	1.6058	5.9937E-07	6.2223	Up
	glutathione S-transferase Mu 3	0.044007	-4.5061	7.1802E-07	6.1439	Down
	beta-1,4-glucuronyltransferase 1	86.879	6.4409	7.4491E-07	6.1279	Up
	protein S100-A6	0.07446	-3.7474	8.8735E-07	6.0519	Down
	cytosolic 10-formyltetrahydrofolate dehydrogenase isoform 1	0.032012	-4.9652	8.925E-07	6.0494	Down
	attractin isoform 1 preproprotein	0.078905	-3.6637	9.0458E-07	6.0436	Down
	beta-glucuronidase isoform 1 precursor	37.256	5.2194	9.1022E-07	6.0409	Up
	protein-arginine deiminase type-4	0.030522	-5.034	9.1338E-07	6.0393	Down
	T-complex protein 1 subunit eta isoform a	0.019121	-5.7087	9.3977E-07	6.027	Down
	alpha-actinin-1 isoform X9	66.38	6.0527	9.6222E-07	6.0167	Up
	small proline-rich protein 2D	45.386	5.5042	1.2694E-06	5.8964	Up
	sulfhydryl oxidase 1 isoform a precursor	0.054095	-4.2084	1.2694E-06	5.8964	Down
	ERO1-like protein alpha isoform 2 precursor	0.022387	-5.4812	1.5358E-06	5.8137	Down
	trefoil factor 2 precursor	104.77	6.711	0.000001583	5.8005	Up
	bactericidal permeability-increasing protein precursor	0.039354	-4.6673	0.000001583	5.8005	Down
	glucose-6-phosphate 1-dehydrogenase isoform a	0.032091	-4.9617	0.000001636	5.7862	Down
	myeloblastin precursor	0.0017668	-9.1447	1.7602E-06	5.7544	Down
	progranulin precursor	29.754	4.895	2.2305E-06	5.6516	Up
	suprabasin isoform X1	0.022789	-5.4555	3.1111E-06	5.5071	Down
	F-box only protein 50	23.448	4.5514	3.2917E-06	5.4826	Up
	contactin-associated protein-like 3B precursor	25.37	4.6651	3.7357E-06	5.4276	Up

	carcinoembryonic antigen-related cell adhesion molecule 5 isoform 1 preproprotein	0.023207	-5.4293	6.9878E-06	5.1557	Down
	peptidase inhibitor 16 precursor	47.274	5.563	0.000010818	4.9658	Up
	cysteine-rich C-terminal protein 1 isoform X1	24.244	4.5996	0.000012922	4.8887	Up
	guanine nucleotide-binding protein G(I)/G(S)/G(T) subunit beta-1 isoform X1	18.045	4.1736	0.00001563	4.806	Up
	elongation factor 1-beta	15.277	3.9333	0.00001563	4.806	Up
	U2 small nuclear ribonucleoprotein B"	23.833	4.5749	0.000021945	4.6587	Up
	glutathione hydrolase 6 isoform X2	18.036	4.1728	0.000023467	4.6296	Up
	lithostathine-1-alpha precursor	59.408	5.8926	0.000028081	4.5516	Up
	epidermal growth factor receptor kinase substrate 8-like protein 1 isoform X1	12.921	3.6917	0.000029948	4.5236	Up
	rho GDP-dissociation inhibitor 1 isoform e	14.325	3.8405	0.000030653	4.5135	Up
	vascular cell adhesion protein 1 isoform a precursor	19.152	4.2594	0.000034881	4.4574	Up
	homeobox protein GBX-1	12.802	3.6783	0.000040465	4.3929	Up
	transitional endoplasmic reticulum ATPase isoform 1	36.097	5.1738	0.000041849	4.3783	Up
	ATP-dependent RNA helicase DDX3X isoform 1	13.682	3.7743	0.000041849	4.3783	Up
	ATP-dependent translocase ABCB1 isoform 1	19.589	4.292	0.000045729	4.3398	Up
	tumor protein p63-regulated gene 1 protein isoform X1	19.589	4.292	0.000045729	4.3398	Up
	glutaredoxin-3 isoform 1	12.769	3.6746	0.000068219	4.1661	Up
	carboxypeptidase N subunit 2 precursor	26.968	4.7532	0.00007362	4.133	Up
	hemicentin-1 precursor	19.791	4.3068	0.000084609	4.0726	Up
	nipped-B-like protein isoform A	10.436	3.3835	0.000089925	4.0461	Up
	interleukin-1 receptor antagonist protein isoform 2	20.575	4.3628	0.000097325	4.0118	Up
	beta-2-glycoprotein 1 precursor	11.448	3.517	0.00010658	3.9723	Up
	peptidyl-prolyl cis-trans isomerase FKBP4	25.31	4.6616	0.00010711	3.9702	Up
	dipeptidyl peptidase 4 isoform 1	28.985	4.8572	0.00012372	3.9076	Up
	zinc finger protein 185 isoform X7	12.689	3.6655	0.00012743	3.8947	Up
	fibrinogen beta chain isoform 1 preproprotein	62.29	5.9609	0.00013148	3.8812	Up
	protein S100-A2 isoform 1	15.783	3.9803	0.00014846	3.8284	Up
	actin-related protein 3 isoform 1	10.343	3.3706	0.00014846	3.8284	Up
	rabphilin-3A isoform 1	12.861	3.6849	0.00017188	3.7648	Up

	hairy and enhancer of split-related protein HELT isoform X1	142.22	7.152	0.00018562	3.7314	Up
	neutral alpha-glucosidase AB isoform 3 precursor	57.271	5.8397	0.00018886	3.7239	Up
	alpha-1B-glycoprotein precursor	4.2215	2.0777	0.00020194	3.6948	Up
	osteoclast-associated immunoglobulin-like receptor isoform 1 precursor	17.724	4.1476	0.0002035	3.6914	Up
	destrin isoform a	17.175	4.1022	0.0002355	3.628	Up
	major prion protein preproprotein Prp precursor	11.116	3.4745	0.00025826	3.5879	Up
	serine/threonine-protein kinase MRCK alpha isoform F	10.063	3.331	0.00026083	3.5836	Up
	N(G),N(G)-dimethylarginine dimethylaminohydrolase 2 isoform X1	20.713	4.3725	0.00026584	3.5754	Up
	corticosteroid-binding globulin isoform X1	35.75	5.1599	0.00027676	3.5579	Up
	TBC1 domain family member 8 isoform 1	23.889	4.5783	0.00028586	3.5439	Up
	peroxiredoxin-5, mitochondrial isoform L precursor	3.831	1.9377	0.00028586	3.5439	Up
	nectin-2 isoform delta precursor	15.468	3.9512	0.00028793	3.5407	Up
	general vesicular transport factor p115 isoform X1	30.466	4.9291	0.0002889	3.5392	Up
	ras-related protein Rab-7a	12.569	3.6517	0.00029588	3.5289	Up
	heterogeneous nuclear ribonucleoproteins A2/B1 isoform X1	13.646	3.7704	0.0003565	3.4479	Up
	enoyl-CoA hydratase, mitochondrial	7.3817	2.884	0.0003565	3.4479	Up
	N-acetyl-D-glucosamine kinase isoform 1	6.5993	2.7223	0.00035799	3.4461	Up
	malate dehydrogenase, mitochondrial isoform 1 precursor	8.4373	3.0768	0.00039704	3.4012	Up
	malate dehydrogenase, cytoplasmic isoform 2	7.0746	2.8226	0.00040807	3.3893	Up
	vinculin isoform meta-VCL	12.588	3.654	0.00041967	3.3771	Up
	perilipin-3 isoform 1	14.45	3.853	0.0004326	3.3639	Up
	aldehyde dehydrogenase, mitochondrial isoform 1 precursor	9.5288	3.2523	0.00045401	3.3429	Up
	elongation factor 1-gamma	28.351	4.8253	0.00045787	3.3393	Up
	neuroblast differentiation-associated protein AHNAK isoform X2	2.0438	1.0313	0.00047078	3.3272	Up
	arf-GAP with Rho-GAP domain, ANK repeat and PH domain-containing protein 1 isoform c	65.864	6.0414	0.00048491	3.3143	Up

	N-acetylglucosamine-6-sulfatase precursor	15.591	3.9626	0.00049833	3.3025	Up
	collagen alpha-2(I) chain precursor	29.94	4.904	0.00050337	3.2981	Up
	15-hydroxyprostaglandin dehydrogenase [NAD(+)] isoform 1	14.789	3.8864	0.00050932	3.293	Up
	plakophilin-3 isoform PKP3b	7.06	2.8197	0.00050932	3.293	Up
	N-sulphoglucosamine sulphohydrolase isoform 1 precursor	11.749	3.5545	0.00052957	3.2761	Up
	delta-1-pyrroline-5-carboxylate dehydrogenase, mitochondrial isoform a precursor	19.026	4.2499	0.00055841	3.253	Up
	heterogeneous nuclear ribonucleoprotein A1 isoform b	14.864	3.8938	0.00056846	3.2453	Up
	40S ribosomal protein S3 isoform 2	10.981	3.457	0.00057	3.2441	Up
	adenine phosphoribosyltransferase isoform a	8.4773	3.0836	0.00057	3.2441	Up
	transforming protein RhoA isoform 1 precursor	14.144	3.8221	0.00057234	3.2423	Up
	prostatic acid phosphatase isoform PAP precursor	6.8413	2.7743	0.00059273	3.2271	Up
	plasminogen activator inhibitor 2	8.8257	3.1417	0.00062782	3.2022	Up
	receptor of activated protein C kinase 1	8.746	3.1286	0.00063876	3.1947	Up
	renin receptor precursor	15.101	3.9166	0.00065936	3.1809	Up
	apolipoprotein E isoform a precursor	19.245	4.2664	0.00066897	3.1746	Up
	ras-related protein Rab-5A isoform 1	10.136	3.3414	0.00068321	3.1654	Up
	alpha-1-acid glycoprotein 1 precursor	4.9356	2.3032	0.00071208	3.1475	Up
	latent-transforming growth factor beta-binding protein 2 precursor	23.298	4.5421	0.00071697	3.1445	Up
	CDK5 regulatory subunit-associated protein 3 isoform c	17.34	4.116	0.00071697	3.1445	Up
	peptidyl-prolyl cis-trans isomerase B precursor	9.5225	3.2513	0.00071697	3.1445	Up
	sialate O-acetylesterase isoform 1 precursor	13.747	3.7811	0.00072742	3.1382	Up
	inorganic pyrophosphatase	12.919	3.6914	0.00072742	3.1382	Up
	dystroglycan 1 isoform X1	17.372	4.1187	0.00073811	3.1319	Up
	60S ribosomal protein L4	20.115	4.3302	0.00084169	3.0748	Up
	complement factor D isoform 2 precursor	20.501	4.3576	0.00086857	3.0612	Up
	eIF5-mimic protein 2 isoform 2	23.84	4.5753	0.00086935	3.0608	Up

	glutathione reductase, mitochondrial isoform 1 precursor	43.834	5.454	0.00091335	3.0394	Up
	beta/gamma crystallin domain-containing protein 1 isoform X1	22.254	4.476	0.00092147	3.0355	Up
	apolipoprotein A-II preproprotein	63.559	5.99	0.00098397	3.007	Up
	zinc-alpha-2-glycoprotein precursor	4.0744	2.0266	0.0010033	2.9986	Up
	bone marrow proteoglycan isoform 1 preproprotein	37.475	5.2279	0.0010697	2.9707	Up
	rho GDP-dissociation inhibitor 2	54.357	5.7644	0.0012219	2.913	Up
	chloride intracellular channel protein 3	4.4697	2.1602	0.0018519	2.7324	Up
	serpin B13 isoform 1	2.7981	1.4845	0.0024827	2.6051	Up
	heat shock protein HSP 90-alpha isoform 1	3.4792	1.7988	0.0051535	2.2879	Up
	plakophilin-1 isoform 1b	4.1988	2.07	0.0056845	2.2453	Up
	plastin-3 isoform X1	0.14354	-2.8005	0.0062497	2.2041	Down
	leukocyte-associated immunoglobulin-like receptor 1 isoform a precursor	2.4936	1.3182	0.0062497	2.2041	Up
	moesin isoform X1	6.6435	2.7319	0.0072633	2.1389	Up
	glycogen phosphorylase, liver form isoform 1	2.1624	1.1126	0.0094602	2.0241	Up
	multimerin-2 precursor	2.4307	1.2813	0.013267	1.8772	Up
	cell division control protein 42 homolog isoform 1 precursor	3.5596	1.8317	0.01791	1.7469	Up
	betaine--homocysteine S-methyltransferase 1	0.15949	-2.6484	0.021713	1.6633	Down
	guanine nucleotide-binding protein G(I)/G(S)/G(T) subunit beta-2	0.24574	-2.0248	0.023343	1.6318	Down
	periplakin	2.0953	1.0671	0.024641	1.6083	Up
	puromycin-sensitive aminopeptidase isoform 1	0.35581	-1.4908	0.025348	1.5961	Down
	alpha-1-antitrypsin precursor	2.2736	1.185	0.03789	1.4215	Up
	secreted and transmembrane protein 1 precursor	2.0067	1.0048	0.040593	1.3915	Up
	glutamine synthetase	2.7816	1.4759	0.041377	1.3832	Up
	endosialin precursor	0.15691	-2.672	0.048945	1.3103	Down

Table S5. Statistics of top 10 enriched gene and KEGG pathways related to DEP.

GO category	GO term	#Matched genes	Percentage of proteins (%)	p-value	Hits	Fold enrichment	FDR
**Biological processes**	GO:0010951~negative regulation of endopeptidase activity	11	3.1884	1.38E-08	51	12.472	3.15E-05
	GO:0045087~innate immune response	28	8.1159	5.33E-07	528	3.067	4.28E-04
	GO:0042730~fibrinolysis	7	2.0290	5.64E-07	19	21.304	4.28E-04
	GO:0002181~cytoplasmic translation	11	3.1884	3.42E-06	90	7.068	1.95E-03
	GO:0050821~protein stabilization	16	4.6377	1.11E-05	229	4.040	5.06E-03
	GO:1904851~positive regulation of establishment of protein localization to telomere	5	1.4493	1.68E-05	10	28.913	6.37E-03
	GO:1904871~positive regulation of protein localization to Cajal body	5	1.4493	2.60E-05	11	26.284	7.60E-03
	GO:0032212~positive regulation of telomere maintenance via telomerase	7	2.0290	2.67E-05	35	11.565	7.60E-03
	GO:0006953~acute-phase response	7	2.0290	5.88E-05	40	10.120	1.49E-02
	GO:0006749~glutathione metabolic process	7	2.0290	6.79E-05	41	9.873	1.54E-02
**Cellular components**	GO:0070062~extracellular exosome	260	75.3623	2.79E-180	2241	7.0025	1.04E-177
	GO:0005576~extracellular region	115	33.3333	7.24E-31	2172	3.1956	1.35E-28
	GO:0005829~cytosol	180	52.1739	2.25E-24	5537	1.9621	2.79E-22
	GO:0005615~extracellular space	97	28.1159	8.71E-24	1931	3.0319	8.10E-22
	GO:0035578~azurophil granule lumen	23	6.6667	7.54E-20	91	15.2548	5.61E-18
	GO:0072562~blood microparticle	27	7.8261	1.45E-19	147	11.0858	8.96E-18
	GO:1904813~ficolin-1-rich granule lumen	25	7.2464	5.04E-19	125	12.0712	2.68E-17
	GO:0005925~focal adhesion	37	10.7246	1.15E-15	428	5.2177	5.34E-14
	GO:0005737~cytoplasm	161	46.6667	4.88E-15	5636	1.7241	2.02E-13
	GO:0034774~secretory granule lumen	19	5.5072	6.94E-13	116	9.8859	2.58E-11

**Molecular functions**	GO:0045296~cadherin binding	37	10.7246	6.71E-19	320	6.5175	4.46E-16
	GO:0005509~calcium ion binding	41	11.8841	4.31E-10	746	3.0979	1.43E-07
	GO:0050839~cell adhesion molecule binding	13	3.7681	1.93E-09	67	10.9369	4.28E-07
	GO:0042802~identical protein binding	61	17.6812	3.23E-07	1734	1.9829	5.37E-05
	GO:0005515~protein binding	266	77.1014	6.01E-07	12707	1.1800	7.99E-05
	GO:0005201~extracellular matrix structural constituent	13	3.7681	1.23E-06	118	6.2099	1.36E-04
	GO:0051015~actin filament binding	17	4.9275	1.50E-06	215	4.4569	1.43E-04
	GO:0003723~RNA binding	52	15.0725	2.92E-06	1475	1.9872	2.42E-04
	GO:0004867~serine-type endopeptidase inhibitor activity	12	3.4783	3.37E-06	108	6.2630	2.49E-04
	GO:0002020~protease binding	12	3.4783	5.25E-06	113	5.9859	3.49E-04
